# Upstrapping to determine futility: predicting future outcomes nonparametrically from past data

**DOI:** 10.1186/s13063-024-08136-3

**Published:** 2024-05-09

**Authors:** Jessica L. Wild, Adit A. Ginde, Christopher J. Lindsell, Alexander M. Kaizer

**Affiliations:** 1 Department of Biostatistics and Informatics, Colorado School of Public Health, University of Colorado Anschutz Medical Campus, Aurora, CO USA; 2 Department of Emergency Medicine, University of Colorado School of Medicine, Aurora, CO USA; 3 Department of Biostatistics and Bioinformatics, School of Medicine, Duke University, Durham, NC USA

**Keywords:** Interim monitoring, Futility monitoring, Upstrap, Nonparametric, Alpha-spending, Conditional power

## Abstract

**Background:**

Clinical trials often involve some form of interim monitoring to determine futility before planned trial completion. While many options for interim monitoring exist (e.g., alpha-spending, conditional power), nonparametric based interim monitoring methods are also needed to account for more complex trial designs and analyses. The upstrap is one recently proposed nonparametric method that may be applied for interim monitoring.

**Methods:**

Upstrapping is motivated by the case resampling bootstrap and involves repeatedly sampling with replacement from the interim data to simulate thousands of fully enrolled trials. The *p*-value is calculated for each upstrapped trial and the proportion of upstrapped trials for which the *p*-value criteria are met is compared with a pre-specified decision threshold. To evaluate the potential utility for upstrapping as a form of interim futility monitoring, we conducted a simulation study considering different sample sizes with several different proposed calibration strategies for the upstrap. We first compared trial rejection rates across a selection of threshold combinations to validate the upstrapping method. Then, we applied upstrapping methods to simulated clinical trial data, directly comparing their performance with more traditional alpha-spending and conditional power interim monitoring methods for futility.

**Results:**

The method validation demonstrated that upstrapping is much more likely to find evidence of futility in the null scenario than the alternative across a variety of simulations settings. Our three proposed approaches for calibration of the upstrap had different strengths depending on the stopping rules used. Compared to O’Brien-Fleming group sequential methods, upstrapped approaches had type I error rates that differed by at most 1.7% and expected sample size was 2–22% lower in the null scenario, while in the alternative scenario power fluctuated between 15.7% lower and 0.2% higher and expected sample size was 0–15% lower.

**Conclusions:**

In this proof-of-concept simulation study, we evaluated the potential for upstrapping as a resampling-based method for futility monitoring in clinical trials. The trade-offs in expected sample size, power, and type I error rate control indicate that the upstrap can be calibrated to implement futility monitoring with varying degrees of aggressiveness and that performance similarities can be identified relative to considered alpha-spending and conditional power futility monitoring methods.

**Supplementary Information:**

The online version contains supplementary material available at 10.1186/s13063-024-08136-3.

## Background

Interim futility monitoring is an essential part of many clinical trial designs. In addition to providing opportunities for safety monitoring and more effectively allocating patients to the most effective treatment possible, interim monitoring can also increase efficiency by stopping trials that show particularly strong signs of intervention futility before their planned endpoints and target sample size [1]. This allows time and resources to be directed towards interventions that show promising results early on in the process and away from interventions that are likely to yield null or underpowered results at the end of the trial. Interim monitoring is commonly used in trials of many different designs across all phases of the research process, with multiple monitoring points allowing the potential to stop a trial early at several points before full data collection is complete. Interim monitoring should always be accounted for at the design stage of a trial to avoid an inflated type I error rate or a reduction in statistical power [2–4].

There are many methods available to perform interim monitoring, including group sequential designs and alpha-spending functions with boundaries such as O’Brien-Fleming, Peto, or Pocock [2–8]. Group sequential designs generate *p*-value boundaries for futility to be applied at the planned monitoring points and at conclusion of the trial [1, 8]. These designs also account for multiple interim monitoring points to maintain the desired trial operating characteristics, such as power and the type I error rate.

Another method used for futility monitoring is conditional power. Conditional power approaches rely on extrapolating the likelihood of finding a positive result at trial completion given the interim data [1, 9]. Like group sequential designs, conditional power can be used to define interim stopping boundaries at pre-planned analysis points, while controlling the power and type I error rate. After calculating conditional power from the interim dataset this can then be compared to a pre-specified stopping boundary to determine whether the trial should stop early (e.g., declare futility if the conditional power is less than 10% based on interim data) [10].

One recently proposed nonparametric framework that could be applied in the context of interim monitoring is the *upstrap*, a resampling-based strategy that builds on the concept of the case resampling bootstrap [11]. Upstrapping relies on repeatedly resampling incomplete data to impute future observations. In a clinical trial context, this could be applied to predict chances of trial success, similar to conditional power or the Bayesian posterior predictive probability of success. The method has been implemented to perform interim monitoring in clinical trials [12–14]. However, there has not yet been a thorough review of the upstrap method’s performance or validity when used for interim monitoring.

In this paper, we seek to fill the evidence gap of the potential for the upstrap as a futility interim monitoring method by evaluating its performance under a more straight-forward binary outcome. We use simulation studies to evaluate the general performance of the upstrap algorithm when used for interim monitoring, which has readily available alpha-spending and conditional power approaches for comparison. In the “Methods” section we introduce the concept of the upstrap as applied to interim monitoring in clinical trials and potential calibration strategies to identify stopping rules. The “Results” section presents the results from our simulation studies, which are used to elucidate the properties of the upstrap and compare to existing methods for interim monitoring. We conclude in the “Case study: TREAT NOW data application” section with a discussion of this newly proposed approach to interim monitoring and settings where the proposed calibrations may be appropriate.

## Methods

### Upstrap algorithm

Upstrapping is a generic approach inspired by bootstrap resampling that resamples the available data (with replacement) to supplement data already collected until a new dataset is generated that matches a desired total sample size [11]. In the context of a clinical trial, this represents the planned maximum sample size to enroll assuming no early termination at an interim analysis. The resampling is done within each treatment group to preserve the desired allocation ratio (e.g., 1:1 between study arms).

The steps for applying upstrapping to an interim analysis dataset are:

(i) Resample with replacement from the observed interim data up to the expected total enrollment to generate a “complete” dataset.
(ii) Calculate the* p*-value for the upstrapped “complete” dataset using the intended final analysis method.
(iii) Repeat a large number of times (e.g., $N_{U_p}=1000$).
(iv) Calculate the proportion of upstrapped *p*-values that meet a set* p*-value threshold (e.g., $p < 0.05$).
(v) Compare the calculated proportion to the set proportion threshold (e.g., $P<0.05$). If the proportion of resampled datasets that meet the given *p*-value threshold is less than *P*, declare trial futility.

To better illustrate this process, consider the following example: a trial is designed with an upstrapping-based interim futility analysis at the 50% stopping point. Using 1000 upstrapped datasets, only 40 met the *p*-value threshold of $p < 0.05$. This is a proportion of 0.04, which is less than the set proportion threshold of $P<0.05$. Therefore, the upstrapped futility analysis would recommend that this trial stop due to anticipated futility.

Notably, this means that set *p*-value and proportion thresholds must be determined a priori. At any interim stage, the upstrap could be used to estimate the probability that the trial will be “successful” based on the given thresholds, with a decision made with regard to potentially stopping the trial for futility.

### Simulation settings

A variety of simulation settings were considered based on three varying parameters: sample size, power, and interim analysis monitoring point. Each simulation setting included a binary outcome measured once per subject with subjects assigned to either treatment or control. For purposes of this simulation, we assume all outcome data is collected at enrollment without missingness or censoring. The proportion of patients with a positive outcome in the control group was set to be 0.6, while the proportion in the treatment group was calibrated to maintain 80% power and a 5% type I error rate for the fixed sample design. The maximum sample size for each treatment group was selected to reflect a range of trial sizes: 20, 80, 300, and 1000 (corresponding to total sample sizes of 40, 160, 600, and 2000). Power includes cases representing an 80% power alternative scenario and 5% type I error rate null scenario, as well as underpowered (50% power) and overpowered (95% power) scenarios to reflect real world uncertainty. Interim monitoring points were based on proportion of subjects enrolled (0.25, 0.50, or 0.75) and are considered to be sequential within a trial. For each setting, we considered *p*-value thresholds between 0 and 0.1 and proportion thresholds from 0 to 1 when applying the upstrapping algorithm. Chi-squared or Fisher’s exact test, depending on test assumptions, were used to estimate the treatment effect in every upstrapped dataset as well as the full sample dataset. For full sample analyses, a two-sided *p*-value of less than 0.05 was considered to be significant (except for alpha-spending interim monitoring approaches which adjust the full sample *p*-value to control type I error). Upstrapping was performed using $N_{U_p}=1000$ upstrapped datasets at each interim analysis. Each simulation setting was repeated 1000 times using R v4.0.2 (Vienna, Austria).

### Method validation

The first research aim was to validate use of the upstrap method for interim monitoring. This involved using simulation results to evaluate the method’s probability of stopping based on the grid of *p*-value and proportion thresholds (defined as a 20 × 20 grid with *p*-values between 0 and 0.1 and proportions between 0 and 1). This grid of threshold values was applied to each simulation setting, providing results across all combinations of sample size, power, and proportion of subjects enrolled. To simplify the validation, the summary figures present the proportion of upstrapped samples which meet the given *p*-value and proportion combination. Essentially, the goal of this research aim is to compare how often the upstrap method determines a trial will stop based on a variety of threshold values, then evaluate how this proportion grid changes between simulation settings.

### Method calibration

Since the upstrap method relies on two key threshold values, *p*-value (*p*) and proportion of upstrapped samples (*P*), we must also consider how to properly calibrate these based on the simulation results. Using the grid of potential *p*-value and proportion threshold values discussed in the “Method validation” section, three different calibration approaches were considered and are presented in the following paragraphs.

***Arbitrary calibration (AU)*** assumes the desired alpha-level for the *p*-value threshold assuming the proportion of upstrapped samples less than the desired type I error rate. We chose $p<0.05$ and $P<0.05$ for the *p*-value and proportion threshold criteria.

***Variable calibration (CU)*** uses a pre-specified grid of potential *p*-value and proportion thresholds and then identifies the *p*-value and proportion threshold combination needed for futility monitoring to achieve a desired level of type I error rate or power.

***Group sequential inspired calibration (GU)*** uses the *p*-values from the alpha-spending O’Brien-Fleming boundary for the *p*-value threshold and searches for a corresponding proportion to achieve a desired level of type I error or power.

For each calibration approach, the rejection rates were calculated (i.e., type I error rate for null scenarios, power for alternative scenarios). Threshold values were then chosen based on attempting to optimize these characteristics, considering all possible threshold combinations without preference. Only threshold combinations producing a type II error rate of at most 20% were considered, then the combination which maximized power was selected from these candidates. This process was done separately for each sample size and monitoring point within the CU and GU calibration approaches.

### Method application

To evaluate the performance of the upstrap algorithm, we applied both upstrapping and group sequential methods to the simulation results to perform interim monitoring. We considered the three different calibration approach described previously, as well as alpha-spending functions with O’Brien-Fleming (OBF) and Pocock (PO) style boundaries. We implemented conditional power approaches using the longCART package in R [15]. We used four different conditional power based stopping boundaries: declaring futility if conditional power was less than 1%, 5%, 10%, or 20%. These four approaches represent a range between more conservative (CP 1%) and more aggressive (CP 20%) stopping boundaries [16]. Since this analysis is focusing specifically on futility monitoring, we exclusively considered futility only monitoring designs and did not also attempt to cover designs involving efficacy monitoring. Interim monitoring points of 0.25, 0.50, and 0.75 were used sequentially within each simulated trial.

Each simulation setting is summarized by the mean and standard deviation of expected sample size, the proportion of trials that stopped early, and the proportion of trials that rejected the null hypothesis. A fixed sample (FS) design without interim monitoring was also implemented to serve as a reference for each design with interim monitoring.

## Results

### Performance of upstrapping across sample sizes and information fractions

Before applying a specific calibration strategy (e.g., AU, CU, or GU), we first evaluated the general trends for sample sizes and information fractions across a grid of *p*-values and proportion of upstrapped samples less than or equal to that *p*-value. This allows for a validation of the general concept of upstrapping, which we present for both the null and alternative scenario.

**Fig. 1:**
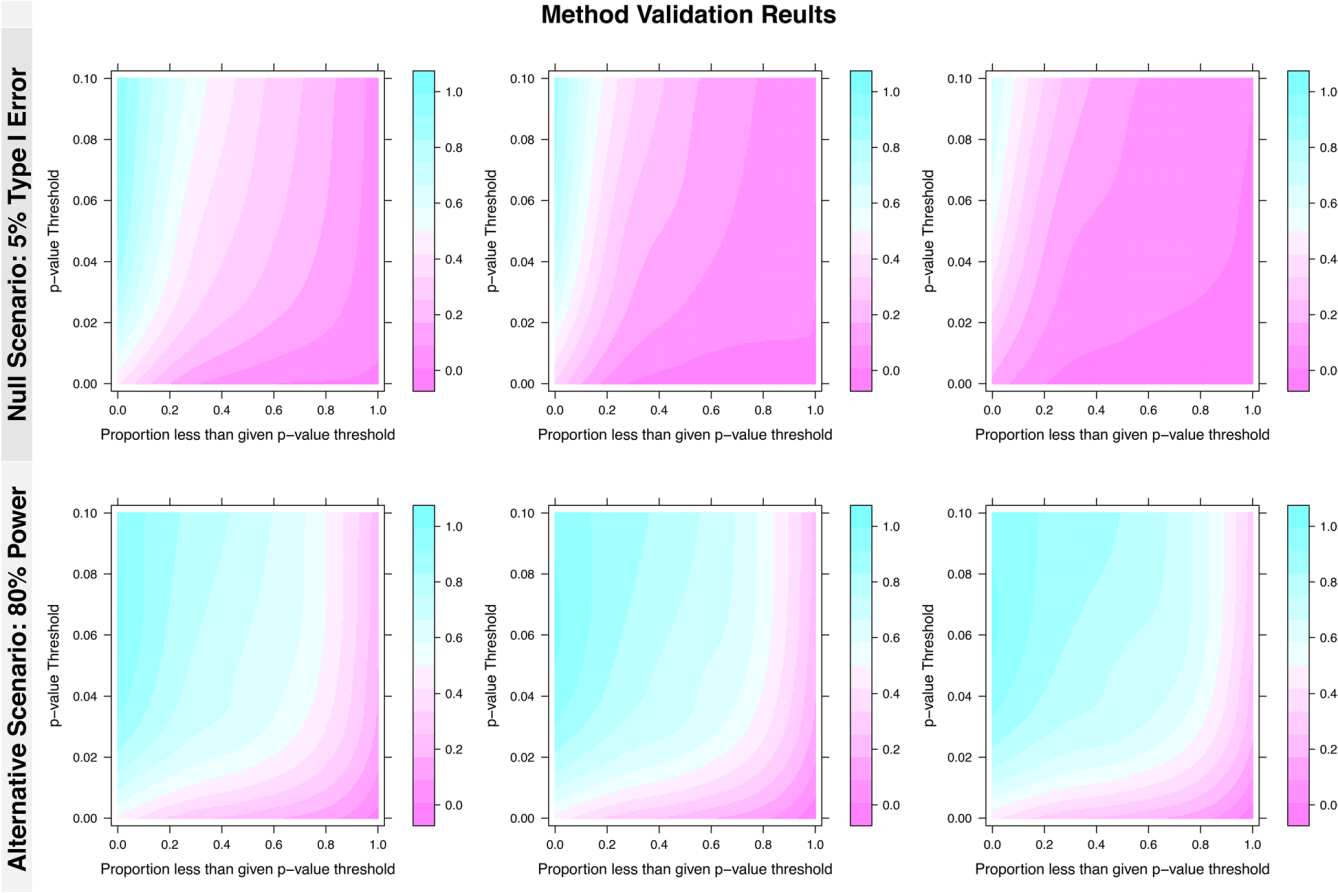
*Method validation results:* Results reported as heatmaps showing the probability of meeting the defined *p*-value and proportion combination (pink representing more likely to meet the criteria for declaring futility, blue representing less likely) for various *p*-value (*y* axis) and proportion (*x* axis) threshold combinations. The null (5% type I error, shown in the top panel) and alternative (80% power, shown in the bottom panel) scenarios are presented with subplots faceted by information fraction at the interim look (0.25, 0.50, 0.75 from left to right). Results are shown for the total sample size equal to 600 setting (results for simulations with total sample sizes of 40, 160, and 2000 are available in the supplementary materials and were not found to vary significantly from the $N = 600$ setting)

Figure 1 displays heatmaps representing the probability of stopping the trial for futility based on the grid of threshold combinations, with different plots showing potential variation due to sample size, power, and the information fraction available at the interim look. Results indicate that across sample sizes and information fractions the upstrap has a higher proportion of upstrapped samples meeting the criteria across different combinations in the null (5% power) case rather than the alternative (80% power) case. This can be thought of as interim monitoring for potential futility, where the higher proportions (i.e., greater pink shaded regions) in the null scenario indicate the upstrap is more likely to meet criteria for potential stopping. These findings are encouraging and indicate that the method produces expected results when used to perform interim futility monitoring. Additionally, results show similar trends across monitoring points within a given sample size, as well as between sample sizes at the same monitoring point. Overall, the validation results provide evidence that upstrapping has higher proportions of stopping (i.e., less blue and more pink) in null scenarios and may be useful for futility monitoring depending on the calibration of stopping rules.

### Sequential monitoring results

This section presents the results for the three proposed upstrap calibration approaches (AU, CU, and GU), two alpha-spending functions (PO and OBF), and four conditional power approaches (CP 1%, CP 5%, CP 10%, and CP 20%) when there are three interim looks at 0.25, 0.50, and 0.75 interim fractions of data under both the null and the 80% powered alternative scenarios. Each interim stopping rule starts with the traditional alpha-spending and conditional power results before introducing the results for the upstrap approaches.

**Fig. 2:**
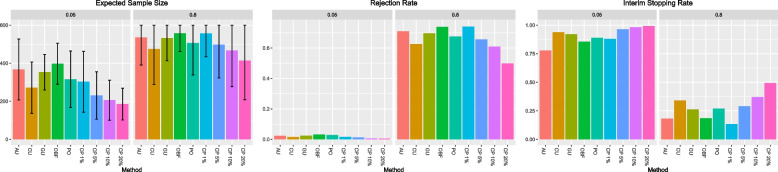
*Main analysis results:* The left panel shows mean expected sample size (*y* axis) reported with error bars representing ± 1 SD for each interim monitoring method (*x* axis). Graphing scale is relative to total sample size. The middle panel shows rejection rate results, where rejection rate is defined as the proportion of simulated trials that reached trial completion and then rejected the null hypothesis. Rejection rate (*y* axis) is reported for each interim monitoring method (*x* axis). The right panel shows interim stopping rate results with interim stopping rate defined as the proportion of simulated trials that stopped early (*y* axis) reported for each interim monitoring method (*x* axis). Subplots are faceted by power (5% or 80% from left to right) and results are shown for the total sample size equal to 600 setting (results for simulations with total sample sizes of 40, 160, and 2000 are available in the supplementary materials and were not found to vary significantly from the $N = 600$ setting)

Figure 2 presents the expected sample size, rejection rates, and interim stopping rates, respectively. Table 1 presents the results for each interim monitoring strategy as the difference in rejection rate from a fixed sample (FS) design without interim monitoring and the ratio of the expected sample size to the fixed sample size for the null and alternative scenarios, respectively. We additionally describe the general performance of the upstrap calibrations when under- or over-powered. The specific numeric summaries of the under- or over-powered scenarios are presented in the Supplementary Materials in Tables S1-S15.

**Table 1:** *Main results* from alternative and null scenario simulations for the performance of the interim analysis calibration method relative to the fixed sample design. CU is the calibrated upstrap, AU is the arbitrary upstrap, GU is the group-sequential upstrap, OBF is the O’Brien-Fleming alpha-spending function, PO is the Pocok alpha-spending function, CP is the conditional power method. ESS refers here to expected sample size, while TIE refers to the type I error rate. Any positive increases in TIE and power for GU, OBF, and PO relative to the fixed sample occur because group sequential designs involve an adjusted *p*-value boundary for the final analysis

		Null scenario		Alternative scenario	
Method	N	Difference in TIE from fixed sample	Ratio of ESS to fixed sample	Difference in power from fixed sample	Ratio of ESS to fixed sample
AU	40	−0.003	0.58	−0.052	0.90
AU	160	−0.008	0.56	−0.072	0.86
AU	600	−0.005	0.61	−0.064	0.89
AU	2000	−0.005	0.62	−0.066	0.91
CU	40	−0.011	0.48	−0.122	0.80
CU	160	−0.009	0.46	−0.135	0.80
CU	600	−0.013	0.45	−0.147	0.79
CU	2000	−0.015	0.45	−0.183	0.79
GU	40	−0.002	0.60	0.001	0.90
GU	160	0.002	0.61	−0.032	0.91
GU	600	−0.004	0.59	−0.076	0.89
GU	2000	−0.008	0.59	−0.083	0.90
OBF	40	−0.002	0.62	−0.001	0.90
OBF	160	0.004	0.64	−0.017	0.92
OBF	600	0.004	0.66	−0.035	0.93
OBF	2000	−0.005	0.67	−0.026	0.94
PO	40	−0.010	0.42	−0.126	0.75
PO	160	0.000	0.49	−0.093	0.82
PO	600	0.000	0.53	−0.097	0.84
PO	2000	−0.001	0.54	−0.081	0.87
CP 1%	40	−0.013	0.52	−0.008	0.98
CP 1%	160	−0.021	0.50	−0.026	0.92
CP 1%	600	−0.012	0.50	−0.033	0.93
CP 1%	2000	−0.019	0.49	−0.047	0.93
CP 5%	40	−0.021	0.42	−0.056	0.90
CP 5%	160	−0.026	0.39	−0.098	0.83
CP 5%	600	−0.016	0.38	−0.117	0.83
CP 5%	2000	−0.025	0.39	−0.122	0.85
CP 10%	40	−0.027	0.38	−0.126	0.85
CP 10%	160	−0.028	0.34	−0.128	0.80
CP 10%	600	−0.022	0.34	−0.164	0.78
CP 10%	2000	−0.031	0.34	−0.203	0.78
CP 20%	40	−0.029	0.32	−0.219	0.72
CP 20%	160	−0.033	0.31	−0.211	0.72
CP 20%	600	−0.024	0.31	−0.275	0.69
CP 20%	2000	−0.035	0.31	−0.296	0.71

#### Main results

The OBF method has type I error rates within 0.5% of the FS design in the null scenario and reductions of approximately 2% to power in the alternative scenario. The ESS across all sample sizes is about 65% of the FS in the null when we expect to stop for futility, and around 92% of the FS in the alternative when stopping for futility is sub-optimal. The PO method has similar type I error rates with even smaller ESS (around 50% of FS under the null), but has approximately a 10% decrease in power relative to the FS design across all sample sizes.

The CP 1% method has type I error rates around 1.6% lower compared to FS, with simultaneous reductions in power of up to 4.7%. ESS is about 50% of the FS in the null case and 94% in the alternative case. As the threshold value used in the CP methods increased from 1 to 20%, the method stops for futility progressively more often, leading to more pronounced decreases in both power and type I error rate relative to the FS design. The most aggressive version of CP (CP 20%) produced decreases in type I error rates of approximately 3% and decreases in power of close to 25%. ESS is around 31% of the FS in the null scenario and 71% in the alternative scenario. CP approaches with thresholds greater than 1% showed undesirably large reductions in power compared to the FS design.

The CU upstrap calibration performs most similarly to the PO, albeit with a greater reduction in power at larger sample sizes of up to 18.3%. The AU and GU upstrap calibrations have type I errors within 1% of the FS design while needing only 56–62% of the ESS. However, the AU power is 5.2–7.2% lower than FS with an ESS of 86–91% of FS, and the GU difference in power increases from within 0.1% of FS to 8.3% lower as the sample size increases.

CP designs are highly influenced by the percentage thresholds chosen but offer significant reductions in sample size, with CP 1% performing best relative to other CP approaches. The OBF is the most balanced with respect to type I error and power trade-offs, but the savings in ESS are minimal relative to the reductions with CP 1%, AU, or GU upstrap calibrations for a futility only monitoring design. If decreasing the expected sample size is an important consideration, AU or GU may represent an acceptable trade-off considering it is about 5% lower than OBF in all null scenarios. AU and GU generally perform comparably to CP 1%, although CP 1% performs moderately better in terms of ESS and power. Additionally, the AU requires no prior calibration, resulting in a low barrier to implementation.

#### Results for under- and over-powered scenarios

Simulations were also conducted to evaluate the performance of the interim monitoring methods if an underpowered (50% power) or overpowered (95% power) scenario were encountered when the design was calibrated assuming an effect size corresponding to 80% power. These results are presented in the supplementary materials with figures and tables showing these scenarios for each of the operating characteristics. The underpowered scenario performs between the previously described null and alternative scenarios. The overpowered scenario has high rejection rates for most methods except PO which has lower power relative to the other approaches.

## Case study: TREAT NOW data application

In order to show how upstrapped futility methods perform when applied to realistic data, we also used data from the previously mentioned TREAT NOW trial as a case study [13, 14]. TREAT NOW examined the use of Lopinavir/ritonavir (administered orally twice a day for 14 days) vs placebo for treating COVID-19 in the outpatient setting. Patients filled out longitudinal surveys asking about the severity of their symptoms each day for study days 1–16, with an additional follow-up survey on study day 28. These results were summarized using a longitudinal ordinal symptom scale as the primary outcome. The original trial was terminated for futility after 75% of the planned sample had been collected. In order to be consistent with the simulation study presented above, we chose to simplify the data into a binary outcome expressing the presence or absence of symptoms at day 15. Multiple imputation was used to handle missing observations. All methods from the simulation study (AU, CU, GU, OBF, PO, CP 1–20%) were then applied to the 25%, 50%, and 75% interim datasets respectively.

Results from the case study are presented in Table 2, showing the results of each futility analysis as well as the futility metric and reference threshold. For upstrapping methods, the futility metric represents the proportion of upstrapped datasets meeting the pre-specified *p*-value threshold *p*, while for OBF and PO the futility metric represents the observed critical value from the interim data, and for conditional power approaches the futility metric represents the calculated power to detect a treatment effect conditional on the interim data. Similarly the reference thresholds for each of these methods convey the proportion threshold, *P*, needed to declare futility (for AU, CU, and GU); the critical value boundary, *Z*, used to define futility using group sequential methods (OBF, PO); or the conditional power threshold, *CP*, used for CP 1–20%. For the upstrapping methods, AU did not stop for futility at any stopping point while CU and GU both declared futility for the 50% and 75% interim looks. It is worth noting however that AU came very close to declaring futility at the 50% stopping point (with a futility metric of 0.065 which was only 0.015 above the threshold of $P < 0.05$) and was also relatively close to declaring futility at the 75% look (with a futility metric of 0.091 which was only 0.041 above the threshold of $P < 0.05$). OBF stopped for futility only at the 75% stopping point, while PO stopped at both 50% and 75%. Of the conditional power approaches only CP 20%, which is the most aggressive implementation of conditional power considered in this analysis, stopped for futility at the 25% and 50% stopping points. CP 20% did not declare futility at the 75% stopping point however. Compared to OBF and conditional power approaches, CU and GU were both able to more quickly and consistently declare futility, leading to gains in trial efficiency. Overall the results of this case study show that, even when applied to realistic clinical datasets, upstrapping methods are a reasonable approach to futility monitoring and may even be able to more efficiently recognize early signs of futility compared to traditional methods.

**Table 2:** *Case study results* from the simplified TREAT NOW data example. For each stage (25%, 50%, and 75% interim looks) and each futility monitoring method, the results are summarized in terms of the futility metric, futility threshold, and the ultimate futility monitoring decision. For upstrapping approaches, futility is determined using the proportion *P*; group sequential approaches determined futility from the critical value *Z*; and conditional power methods determined futility from *CP* which is a measure of the power to detect a treatment effect conditional upon the interim data. OBF determined that it is not possible to stop after the 25% interim look, which means that the reference futility threshold and calculated futility metric are listed as NA for OBF and GU (which utilizes the OBF stopping criteria for its *p*-value threshold *p*). The ultimate decision of the futility analysis is reported as “Continue” for cases where the method decided not to stop early for futility and is reported as “Stop” for cases where the method did decide to stop early for futility

Method	Stage	Calculated futility metric	Reference futility threshold	Futility decision
AU	25%	*P* = 0.515	*P *< 0.05	Continue
AU	50%	*P *= 0.065	*P* < 0.05	Continue
AU	75%	*P* = 0.091	*P *< 0.05	Continue
CU	25%	*P *= 0.549	*P* < 0.20	Continue
CU	50%	*P* = 0.040	*P* < 0.20	Stop
CU	75%	*P *= 0.046	*P* < 0.20	Stop
GU	25%	NA	NA	Continue
GU	50%	*P* = 0.691	*P *< 0.90	Stop
GU	75%	*P* = 0.501	*P* < 0.80	Stop
OBF	25%	NA	NA	Continue
OBF	50%	Z = 0.566	*Z* < 0.543	Continue
OBF	75%	Z = 1.074	*Z* < 1.301	Stop
PO	25%	Z = 0.858	*Z* < 0.280	Continue
PO	50%	Z = 0.566	*Z* < 0.711	Stop
PO	75%	Z = 1.074	*Z* < 1.319	Stop
CP 1%	25%	CP = 0.194	CP < 1%	Continue
CP 1%	50%	CP = 0.143	CP < 1%	Continue
CP 1%	75%	CP = 0.446	CP < 1%	Continue
CP 5%	25%	CP = 0.194	CP < 5%	Continue
CP 5%	50%	CP = 0.143	CP < 5%	Continue
CP 5%	75%	CP = 0.446	CP < 5%	Continue
CP 10%	25%	CP = 0.194	CP < 10%	Continue
CP 10%	50%	CP = 0.143	CP < 10%	Continue
CP 10%	75%	CP = 0.446	CP < 10%	Continue
CP 20%	25%	CP = 0.194	CP < 20%	Stop
CP 20%	50%	CP = 0.143	CP < 20%	Stop
CP 20%	75%	CP = 0.446	CP < 20%	Continue

## Discussion

There are numerous strategies for conducting interim monitoring within a clinical trial. In this paper, we proposed the use of the non-parametric upstrap as a potential futility interim monitoring strategy and evaluated its potential performance across a range of calibration strategies. While alpha-spending and conditional power approaches are well-established methods for interim futility monitoring, both may be limited in some contexts by their assumptions. Group sequential designs are based on Brownian motion which can be thought of as a Gaussian process [8], whereas the conditional power designs implemented in this work rely on normal approximations [9]. While these assumptions may be appropriate for many data types and modeling strategies, there may be settings where nonparametric alternatives would be desired.

This paper presents simulation results using a simple binary outcome to establish the upstrap’s performance in direct comparison to standard methods, using a setting where the assumptions of these traditional methods are not violated so that the upstrap can be more fairly and directly compared to standard interim monitoring designs. Another potential strength of the upstrap is the flexible nature of its resampling approach. For example, the upstrap can be applied to either frequentist or Bayesian paradigms and is similar in many respects to the concepts of conditional power or predictive probability, but it may make fewer assumptions for the interim monitoring methods regarding asymptotic properties or prior specification. The upstrap may also be useful for more complex methods, such as the Bayesian longitudinal ordinal logistic regression model used in the TREAT NOW clinical trial which used an upstrapping approach to address computational limitations in estimating the Bayesian predictive probability of success [13, 14]. In general, the upstrap can be differentiated from conditional power and predictive probability of success methods because of this greater flexibility. By implementing an upstrapping approach researchers can expand interim futility monitoring to a wider variety of settings where it may not be appropriate or desirable to use normal approximations (as needed to implement the conditional power approaches from this paper’s simulation study) or to specify a specific prior distribution (as required for a predictive power interim analysis).

The validation of the general performance of the upstrap across information fractions and different trial sample sizes confirmed the increased likelihood of rejecting for futility at interim analyses under the null instead of the alternative scenario. Upstrapping approaches were generally more likely to stop a trial early compared to PO and OBF, as shown in Fig. 2. CP 20%, the conditional power method most likely to stop early, had generally lower type I error rates and reduced expected sample size compared to upstrapping methods, but with power significantly lower than the upstrapped results. Less aggressive conditional power methods performed better overall, with CP 1% achieving moderately lower expected sample size under the null setting compared to AU, while simultaneously maintaining slight advantages in power and type I error rates. Across all interim futility monitoring settings, the GU approach provided what may be considered as acceptable trade-offs in certain trial contexts considering the reductions in ESS relative to OBF of up to 8% in the null scenario, while having less inflation of the type I error rate than other calibration strategies. The CU approach was overly conservative for futility monitoring. Interestingly, the AU approach performed well in futility monitoring and could be an easy-to-implement approach if slightly lower power is acceptable or the maximum sample size could be increased. Limiting interim analyses to occur later on in the trial, after accumulating a reasonable amount of data from which to make early stopping decisions, may be another effective way to further improve upstrapping approaches.

Calibration and application results showed a clear trade off between power and type I error rate for the general upstrapping method. This is not unexpected, but is an important consideration when deciding on an interim monitoring method and choosing threshold values. In general, we considered several different approaches to threshold calibration and chose the best values based on power and type I error rate considerations. However, this process could easily be extended to consider different approaches or a more granular grid of potential threshold values. Additional work is needed to develop calibration approaches for the upstrap which can better control the type I error rate while achieving the desired power. One possibility is to consider similarities with Bayesian interim monitoring using the predictive posterior probability (PPP), where the posterior probability may be analogous to the *p*-value and the PPP threshold analogous to our upstrapped proportion [17–19].

While this research is a proof-of-concept study of whether upstrapping may be a potential approach for interim monitoring for futility, there are limitations worth discussing. First, many simplifying assumptions were made at both the simulation and modeling stages of our analysis. We considered simulation settings based only on sample size, information fraction, and power for binary outcomes. Further, all simulations assumed uniform subject accrual over time, and a constant treatment efficacy rate. It would be worth considering more complicated modeling strategies and different outcome types, potentially with covariate information included, to reflect a wider range of possible study design contexts.

Based on our simulation studies, the upstrap has potential to serve as a nonparametric approach to implementing interim analyses for futility in clinical trials. Future extensions of this work will focus on applying the upstrap to additional outcome types and more complex trial designs with comparison to existing interim monitoring strategies.

### Supplementary information

**Supplementary Material 1**. Supplementary Tables and Supplementary Figures (referenced in the main text as S1-S15), including results for over and under powered simulation settings and a brief sensitivity analysis on the results of only planning interim monitoring at the 50% and 75% stopping points.

## Data Availability

All supplementary tables and figures, as well as code and simulated datasets, can be found on GitHub at: https://github.com/jessicalynnwild/Upstrap-Futility-Monitoring-Manuscript.

## Abbreviations

OBF: O’Brien-Fleming group sequential design
PO: Pocock group sequential design
AU: Arbitrary upstrap
CU: Calibrated upstrap
GU: Group sequential approach upstrap
TIE: Type I error rate
