# Supplementary material for: Upstrapping to determine futility: predicting future outcomes nonparametrically from past data
Source: Trials. 2024 May 9;25:312. doi: 10.1186/s13063-024-08136-3 (PMC11083808; doi:10.1186/s13063-024-08136-3)
Supplement: Supplementary file 1 — Supplementary Material 1. Supplementary Tables and Supplementary Figures (referenced in the main text as S1-S15), including results for over and under powered simulation settings and a brief sensitivity analysis on the results of only planning interim monitoring at the 50% and 75% stopping points. [file 13063_2024_8136_MOESM1_ESM.html]

Supplementary Materials


# Supplementary Materials

#### Jess L. Wild, MS

#### Alexander M. Kaizer. PhD

#### 04/15/2024

## Tabular Results for Arbitrary Upstrapping (AU)

This section (***Table S1***:***Table
S4***) includes tabular results summaries for the arbitrary
upstrapping (AU) approach to interim monitoring. For each sample size
(\(N\)) and power (\(\beta\)) based scenario three evaluation
metrics are reported: expected total sample size (*Expected \(N\)*), proportion of trials that
stopped early at any interim stopping point (*Proportion
Stopped*), and proportion of trials that either stopped early for
efficacy or rejected the null hypothesis with the full sample data
(*Proportion Rejected*). All tables also include fixed sample
rejection rate results for comparison. ***Table
S1*** reports results for trials designed with a single
interim stopping point at 25% of the total sample size.
***Table S2*** reports results for trials designed
with a single interim stopping point at 50% of the total sample size.
***Table S3*** reports results for trials designed
with a single interim stopping point at 75% of the total sample size.
***Table S4*** reports results for trials designed
with sequential interim stopping points at 25%, 50%, and 75% of the
total sample size.

**Table S1: Simulation Results Using Arbitrary Upstrapping
Thresholds for 0.25 Stopping Point Monitoring**

|  | | ***Futility Only Monitoring*** | | | ***Efficacy Only Monitoring*** | | | ***Futility and Efficacy Monitoring*** | | | | | ***Fixed Sample*** |
| --- | --- | --- | --- | --- | --- | --- | --- | --- | --- | --- | --- | --- | --- |
| N | \(\beta\) | Expected N *mean(sd)* | Proportion Stopped | Proportion Rejected | Expected N *mean(sd)* | Proportion Stopped | Proportion Rejected | Expected N *mean(sd)* | Proportion Stopped | Proportion Stopped (Futility) | Proportion Stopped (Efficacy) | Proportion Rejected | Proportion Rejected |
| 40 | 0.05 | 33 (12.94) | 0.247 | 0.031 | 35 (11.51) | 0.179 | 0.196 | 28 (14.84) | 0.426 | 0.247 | 0.179 | 0.193 | 0.034 |
| 40 | 0.50 | 36 (10.92) | 0.157 | 0.357 | 30 (14.31) | 0.349 | 0.532 | 25 (15.01) | 0.506 | 0.157 | 0.349 | 0.507 | 0.382 |
| 40 | 0.80 | 38 (8.80) | 0.095 | 0.640 | 25 (15.01) | 0.502 | 0.771 | 23 (14.72) | 0.597 | 0.095 | 0.502 | 0.727 | 0.684 |
| 40 | 0.95 | 39 (5.95) | 0.041 | 0.884 | 20 (14.01) | 0.679 | 0.943 | 19 (13.48) | 0.720 | 0.041 | 0.679 | 0.912 | 0.915 |
| 160 | 0.05 | 121 (56.58) | 0.333 | 0.034 | 142 (43.22) | 0.153 | 0.172 | 102 (60.01) | 0.486 | 0.333 | 0.153 | 0.168 | 0.038 |
| 160 | 0.50 | 138 (47.19) | 0.191 | 0.371 | 120 (56.63) | 0.334 | 0.526 | 97 (59.95) | 0.525 | 0.191 | 0.334 | 0.488 | 0.409 |
| 160 | 0.80 | 145 (40.38) | 0.130 | 0.660 | 102 (60.02) | 0.491 | 0.783 | 86 (58.25) | 0.621 | 0.130 | 0.491 | 0.722 | 0.721 |
| 160 | 0.95 | 152 (31.03) | 0.072 | 0.863 | 84 (57.66) | 0.639 | 0.938 | 75 (54.42) | 0.711 | 0.072 | 0.639 | 0.885 | 0.916 |
| 600 | 0.05 | 501 (187.11) | 0.222 | 0.025 | 528 (165.89) | 0.162 | 0.178 | 428 (218.97) | 0.384 | 0.222 | 0.162 | 0.175 | 0.028 |
| 600 | 0.50 | 545 (147.87) | 0.123 | 0.447 | 438 (216.37) | 0.362 | 0.613 | 382 (225.01) | 0.485 | 0.123 | 0.362 | 0.571 | 0.489 |
| 600 | 0.80 | 559 (130.76) | 0.093 | 0.726 | 380 (225.08) | 0.491 | 0.820 | 338 (221.91) | 0.584 | 0.093 | 0.491 | 0.773 | 0.773 |
| 600 | 0.95 | 582 (88.23) | 0.040 | 0.913 | 306 (214.02) | 0.655 | 0.959 | 288 (207.29) | 0.695 | 0.040 | 0.655 | 0.923 | 0.949 |
| 2000 | 0.05 | 1687 (610.20) | 0.209 | 0.039 | 1739 (568.95) | 0.174 | 0.197 | 1426 (729.54) | 0.383 | 0.209 | 0.174 | 0.196 | 0.040 |
| 2000 | 0.50 | 1799 (511.23) | 0.134 | 0.457 | 1459 (720.80) | 0.361 | 0.596 | 1258 (750.34) | 0.495 | 0.134 | 0.361 | 0.561 | 0.492 |
| 2000 | 0.80 | 1886 (397.70) | 0.076 | 0.766 | 1208 (749.20) | 0.528 | 0.859 | 1094 (733.96) | 0.604 | 0.076 | 0.528 | 0.817 | 0.808 |
| 2000 | 0.95 | 1942 (290.54) | 0.039 | 0.932 | 968 (695.31) | 0.688 | 0.969 | 910 (668.59) | 0.727 | 0.039 | 0.688 | 0.941 | 0.960 |

**Table S2: Simulation Results Using Arbitrary Upstrapping
Thresholds for 0.50 Stopping Point Monitoring**

|  | | ***Futility Only Monitoring*** | | | ***Efficacy Only Monitoring*** | | | ***Futility and Efficacy Monitoring*** | | | | | ***Fixed Sample*** |
| --- | --- | --- | --- | --- | --- | --- | --- | --- | --- | --- | --- | --- | --- |
| N | \(\beta\) | Expected N *mean(sd)* | Proportion Stopped | Proportion Rejected | Expected N *mean(sd)* | Proportion Stopped | Proportion Rejected | Expected N *mean(sd)* | Proportion Stopped | Proportion Stopped (Futility) | Proportion Stopped (Efficacy) | Proportion Rejected | Proportion Rejected |
| 40 | 0.05 | 29 (9.90) | 0.572 | 0.034 | 39 (4.44) | 0.052 | 0.074 | 28 (9.69) | 0.624 | 0.572 | 0.052 | 0.074 | 0.034 |
| 40 | 0.50 | 35 (8.70) | 0.253 | 0.367 | 34 (9.18) | 0.301 | 0.457 | 29 (9.95) | 0.554 | 0.253 | 0.301 | 0.442 | 0.382 |
| 40 | 0.80 | 39 (5.81) | 0.093 | 0.672 | 30 (10.00) | 0.503 | 0.734 | 29 (9.82) | 0.596 | 0.093 | 0.503 | 0.722 | 0.684 |
| 40 | 0.95 | 40 (3.06) | 0.024 | 0.908 | 26 (8.84) | 0.734 | 0.936 | 25 (8.57) | 0.758 | 0.024 | 0.734 | 0.929 | 0.915 |
| 160 | 0.05 | 121 (40.02) | 0.495 | 0.037 | 156 (18.71) | 0.058 | 0.081 | 116 (39.79) | 0.553 | 0.495 | 0.058 | 0.080 | 0.038 |
| 160 | 0.50 | 143 (33.32) | 0.223 | 0.393 | 136 (36.71) | 0.301 | 0.478 | 119 (39.97) | 0.524 | 0.223 | 0.301 | 0.462 | 0.409 |
| 160 | 0.80 | 152 (24.22) | 0.102 | 0.703 | 118 (39.96) | 0.527 | 0.768 | 110 (38.67) | 0.629 | 0.102 | 0.527 | 0.750 | 0.721 |
| 160 | 0.95 | 158 (14.91) | 0.036 | 0.905 | 101 (35.15) | 0.739 | 0.930 | 98 (33.42) | 0.775 | 0.036 | 0.739 | 0.919 | 0.916 |
| 600 | 0.05 | 456 (149.98) | 0.482 | 0.024 | 586 (64.79) | 0.049 | 0.069 | 441 (149.79) | 0.531 | 0.482 | 0.049 | 0.065 | 0.028 |
| 600 | 0.50 | 550 (112.48) | 0.169 | 0.464 | 501 (141.24) | 0.331 | 0.549 | 450 (150.08) | 0.500 | 0.169 | 0.331 | 0.524 | 0.489 |
| 600 | 0.80 | 571 (89.24) | 0.098 | 0.748 | 435 (149.29) | 0.551 | 0.797 | 406 (143.26) | 0.649 | 0.098 | 0.551 | 0.772 | 0.773 |
| 600 | 0.95 | 595 (40.98) | 0.019 | 0.937 | 373 (128.37) | 0.759 | 0.959 | 367 (124.74) | 0.778 | 0.019 | 0.759 | 0.947 | 0.949 |
| 2000 | 0.05 | 1555 (497.21) | 0.445 | 0.036 | 1940 (237.61) | 0.060 | 0.084 | 1495 (500.23) | 0.505 | 0.445 | 0.060 | 0.080 | 0.040 |
| 2000 | 0.50 | 1822 (382.70) | 0.178 | 0.480 | 1660 (473.95) | 0.340 | 0.555 | 1482 (499.93) | 0.518 | 0.178 | 0.340 | 0.543 | 0.492 |
| 2000 | 0.80 | 1929 (256.95) | 0.071 | 0.779 | 1444 (497.10) | 0.556 | 0.842 | 1373 (483.84) | 0.627 | 0.071 | 0.556 | 0.813 | 0.808 |
| 2000 | 0.95 | 1983 (129.34) | 0.017 | 0.950 | 1225 (417.79) | 0.775 | 0.966 | 1208 (406.08) | 0.792 | 0.017 | 0.775 | 0.956 | 0.960 |

**Table S3: Simulation Results Using Arbitrary Upstrapping
Thresholds for 0.75 Stopping Point Monitoring**

|  | | ***Futility Only Monitoring*** | | | ***Efficacy Only Monitoring*** | | | ***Futility and Efficacy Monitoring*** | | | | | ***Fixed Sample*** |
| --- | --- | --- | --- | --- | --- | --- | --- | --- | --- | --- | --- | --- | --- |
| N | \(\beta\) | Expected N *mean(sd)* | Proportion Stopped | Proportion Rejected | Expected N *mean(sd)* | Proportion Stopped | Proportion Rejected | Expected N *mean(sd)* | Proportion Stopped | Proportion Stopped (Futility) | Proportion Stopped (Efficacy) | Proportion Rejected | Proportion Rejected |
| 40 | 0.05 | 33 (4.10) | 0.786 | 0.034 | 40 (1.62) | 0.027 | 0.046 | 32 (3.90) | 0.813 | 0.786 | 0.027 | 0.046 | 0.034 |
| 40 | 0.50 | 37 (4.63) | 0.311 | 0.382 | 38 (4.43) | 0.268 | 0.410 | 35 (4.94) | 0.579 | 0.311 | 0.268 | 0.410 | 0.382 |
| 40 | 0.80 | 39 (3.04) | 0.103 | 0.679 | 35 (4.99) | 0.538 | 0.710 | 34 (4.80) | 0.641 | 0.103 | 0.538 | 0.705 | 0.684 |
| 40 | 0.95 | 40 (1.59) | 0.026 | 0.911 | 32 (3.87) | 0.817 | 0.927 | 32 (3.64) | 0.843 | 0.026 | 0.817 | 0.923 | 0.915 |
| 160 | 0.05 | 131 (17.40) | 0.747 | 0.036 | 159 (7.35) | 0.035 | 0.053 | 129 (16.52) | 0.782 | 0.747 | 0.035 | 0.051 | 0.038 |
| 160 | 0.50 | 148 (18.62) | 0.317 | 0.401 | 149 (17.91) | 0.277 | 0.441 | 137 (19.65) | 0.594 | 0.317 | 0.277 | 0.433 | 0.409 |
| 160 | 0.80 | 156 (12.96) | 0.119 | 0.717 | 138 (19.88) | 0.557 | 0.742 | 133 (18.73) | 0.676 | 0.119 | 0.557 | 0.738 | 0.721 |
| 160 | 0.95 | 159 (6.37) | 0.026 | 0.913 | 129 (16.39) | 0.787 | 0.925 | 128 (15.60) | 0.813 | 0.026 | 0.787 | 0.922 | 0.916 |
| 600 | 0.05 | 495 (68.64) | 0.702 | 0.027 | 597 (23.43) | 0.025 | 0.044 | 491 (66.86) | 0.727 | 0.702 | 0.025 | 0.043 | 0.028 |
| 600 | 0.50 | 564 (64.28) | 0.242 | 0.478 | 546 (72.08) | 0.361 | 0.522 | 510 (73.43) | 0.603 | 0.242 | 0.361 | 0.511 | 0.489 |
| 600 | 0.80 | 589 (40.48) | 0.079 | 0.768 | 506 (72.50) | 0.629 | 0.788 | 494 (68.24) | 0.708 | 0.079 | 0.629 | 0.783 | 0.773 |
| 600 | 0.95 | 598 (17.63) | 0.014 | 0.944 | 474 (54.17) | 0.846 | 0.956 | 471 (52.07) | 0.860 | 0.014 | 0.846 | 0.951 | 0.949 |
| 2000 | 0.05 | 1645 (226.76) | 0.711 | 0.039 | 1984 (88.04) | 0.032 | 0.058 | 1629 (218.60) | 0.743 | 0.711 | 0.032 | 0.057 | 0.040 |
| 2000 | 0.50 | 1890 (207.23) | 0.220 | 0.486 | 1819 (240.41) | 0.362 | 0.521 | 1709 (246.74) | 0.582 | 0.220 | 0.362 | 0.515 | 0.492 |
| 2000 | 0.80 | 1967 (125.07) | 0.067 | 0.801 | 1671 (237.14) | 0.659 | 0.830 | 1637 (223.12) | 0.726 | 0.067 | 0.659 | 0.823 | 0.808 |
| 2000 | 0.95 | 1995 (49.77) | 0.010 | 0.959 | 1569 (172.01) | 0.863 | 0.963 | 1564 (166.57) | 0.873 | 0.010 | 0.863 | 0.962 | 0.960 |

**Table S4: Simulation Results Using Arbitrary Upstrapping
Thresholds for Sequential Monitoring**

|  | | ***Futility Only Monitoring*** | | | ***Efficacy Only Monitoring*** | | | ***Futility and Efficacy Monitoring*** | | | | | ***Fixed Sample*** |
| --- | --- | --- | --- | --- | --- | --- | --- | --- | --- | --- | --- | --- | --- |
| N | \(\beta\) | Expected N *mean(sd)* | Proportion Stopped | Proportion Rejected | Expected N *mean(sd)* | Proportion Stopped | Proportion Rejected | Expected N *mean(sd)* | Proportion Stopped | Proportion Stopped (Futility) | Proportion Stopped (Efficacy) | Proportion Rejected | Proportion Rejected |
| 40 | 0.05 | 23 (10.08) | 0.839 | 0.031 | 35 (11.65) | 0.207 | 0.217 | 19 (9.06) | 0.933 | 0.731 | 0.202 | 0.211 | 0.034 |
| 40 | 0.50 | 31 (11.66) | 0.424 | 0.350 | 28 (13.86) | 0.494 | 0.570 | 19 (10.98) | 0.848 | 0.361 | 0.487 | 0.540 | 0.382 |
| 40 | 0.80 | 36 (9.76) | 0.202 | 0.632 | 22 (13.01) | 0.717 | 0.800 | 18 (10.58) | 0.876 | 0.183 | 0.693 | 0.746 | 0.684 |
| 40 | 0.95 | 39 (6.56) | 0.072 | 0.876 | 16 (9.93) | 0.903 | 0.957 | 15 (8.64) | 0.941 | 0.061 | 0.880 | 0.920 | 0.915 |
| 160 | 0.05 | 90 (44.15) | 0.815 | 0.032 | 139 (44.37) | 0.195 | 0.202 | 74 (39.46) | 0.911 | 0.720 | 0.191 | 0.194 | 0.038 |
| 160 | 0.50 | 122 (48.29) | 0.440 | 0.355 | 111 (55.12) | 0.475 | 0.562 | 76 (44.19) | 0.855 | 0.399 | 0.456 | 0.507 | 0.409 |
| 160 | 0.80 | 138 (42.86) | 0.233 | 0.649 | 88 (52.50) | 0.703 | 0.809 | 68 (41.38) | 0.885 | 0.219 | 0.666 | 0.737 | 0.721 |
| 160 | 0.95 | 150 (32.99) | 0.102 | 0.856 | 68 (42.27) | 0.875 | 0.945 | 60 (34.88) | 0.934 | 0.096 | 0.838 | 0.882 | 0.916 |
| 600 | 0.05 | 368 (159.90) | 0.778 | 0.023 | 521 (168.13) | 0.188 | 0.199 | 306 (154.54) | 0.873 | 0.690 | 0.183 | 0.190 | 0.028 |
| 600 | 0.50 | 492 (163.13) | 0.353 | 0.428 | 393 (205.42) | 0.543 | 0.645 | 297 (169.15) | 0.837 | 0.314 | 0.523 | 0.583 | 0.489 |
| 600 | 0.80 | 536 (144.81) | 0.180 | 0.709 | 319 (190.46) | 0.740 | 0.840 | 264 (158.61) | 0.876 | 0.170 | 0.706 | 0.775 | 0.773 |
| 600 | 0.95 | 577 (95.51) | 0.062 | 0.901 | 245 (150.94) | 0.894 | 0.964 | 226 (131.86) | 0.936 | 0.060 | 0.876 | 0.917 | 0.949 |
| 2000 | 0.05 | 1244 (527.49) | 0.778 | 0.035 | 1715 (577.12) | 0.202 | 0.220 | 1015 (503.02) | 0.892 | 0.690 | 0.202 | 0.215 | 0.040 |
| 2000 | 0.50 | 1630 (558.83) | 0.347 | 0.447 | 1310 (684.42) | 0.543 | 0.635 | 979 (566.66) | 0.831 | 0.309 | 0.522 | 0.587 | 0.492 |
| 2000 | 0.80 | 1824 (440.22) | 0.156 | 0.742 | 1020 (624.09) | 0.769 | 0.875 | 866 (520.51) | 0.892 | 0.146 | 0.746 | 0.809 | 0.808 |
| 2000 | 0.95 | 1931 (305.80) | 0.051 | 0.925 | 781 (479.86) | 0.913 | 0.974 | 729 (427.46) | 0.943 | 0.050 | 0.893 | 0.940 | 0.960 |

## Tabular Results for Calibrated Upstrapping (CU)

This section (***Table S5***:***Table
S8***) includes tabular results summaries for the calibrated
upstrapping (CU) approach to interim monitoring. For each sample size
(\(N\)) and power (\(\beta\)) based scenario three evaluation
metrics are reported: expected total sample size (*Expected \(N\)*), proportion of trials that
stopped early at any interim stopping point (*Proportion
Stopped*), and proportion of trials that either stopped early for
efficacy or rejected the null hypothesis with the full sample data
(*Proportion Rejected*). All tables also include fixed sample
rejection rate results for comparison. ***Table
S5*** reports results for trials designed with a single
interim stopping point at 25% of the total sample size.
***Table S6*** reports results for trials designed
with a single interim stopping point at 50% of the total sample size.
***Table S7*** reports results for trials designed
with a single interim stopping point at 75% of the total sample size.
***Table S8*** reports results for trials designed
with sequential interim stopping points at 25%, 50%, and 75% of the
total sample size.

**Table S5: Simulation Results Using Calibrated Upstrapping
Thresholds for 0.25 Stopping Point Monitoring**

|  | | ***Futility Only Monitoring*** | | | ***Efficacy Only Monitoring*** | | | ***Futility and Efficacy Monitoring*** | | | | | ***Fixed Sample*** |
| --- | --- | --- | --- | --- | --- | --- | --- | --- | --- | --- | --- | --- | --- |
| N | \(\beta\) | Expected N *mean(sd)* | Proportion Stopped | Proportion Rejected | Expected N *mean(sd)* | Proportion Stopped | Proportion Rejected | Expected N *mean(sd)* | Proportion Stopped | Proportion Stopped (Futility) | Proportion Stopped (Efficacy) | Proportion Rejected | Proportion Rejected |
| 40 | 0.05 | 27 (14.90) | 0.441 | 0.026 | 40 (0.00) | 0.000 | 0.034 | 27 (14.90) | 0.441 | 0.441 | 0.000 | 0.026 | 0.034 |
| 40 | 0.50 | 31 (14.00) | 0.320 | 0.312 | 40 (0.00) | 0.000 | 0.382 | 31 (14.00) | 0.320 | 0.320 | 0.000 | 0.312 | 0.382 |
| 40 | 0.80 | 35 (11.89) | 0.195 | 0.592 | 40 (0.00) | 0.000 | 0.684 | 35 (11.89) | 0.195 | 0.195 | 0.000 | 0.592 | 0.684 |
| 40 | 0.95 | 38 (7.81) | 0.073 | 0.861 | 40 (0.00) | 0.000 | 0.915 | 38 (7.81) | 0.073 | 0.073 | 0.000 | 0.861 | 0.915 |
| 160 | 0.05 | 106 (59.82) | 0.458 | 0.034 | 155 (25.66) | 0.048 | 0.078 | 100 (60.03) | 0.506 | 0.458 | 0.048 | 0.074 | 0.038 |
| 160 | 0.50 | 127 (53.73) | 0.277 | 0.348 | 142 (42.87) | 0.150 | 0.448 | 109 (59.39) | 0.427 | 0.277 | 0.150 | 0.387 | 0.409 |
| 160 | 0.80 | 138 (47.00) | 0.189 | 0.625 | 129 (52.66) | 0.260 | 0.738 | 107 (59.72) | 0.449 | 0.189 | 0.260 | 0.642 | 0.721 |
| 160 | 0.95 | 146 (39.15) | 0.121 | 0.820 | 113 (58.72) | 0.396 | 0.921 | 98 (60.00) | 0.517 | 0.121 | 0.396 | 0.825 | 0.916 |
| 600 | 0.05 | 394 (224.32) | 0.458 | 0.020 | 579 (96.24) | 0.048 | 0.072 | 373 (225.10) | 0.506 | 0.458 | 0.048 | 0.064 | 0.028 |
| 600 | 0.50 | 479 (200.11) | 0.271 | 0.404 | 529 (164.64) | 0.159 | 0.527 | 407 (222.90) | 0.430 | 0.271 | 0.159 | 0.442 | 0.489 |
| 600 | 0.80 | 511 (179.41) | 0.198 | 0.672 | 484 (197.24) | 0.259 | 0.786 | 395 (224.28) | 0.457 | 0.198 | 0.259 | 0.685 | 0.773 |
| 600 | 0.95 | 555 (135.67) | 0.101 | 0.864 | 426 (219.29) | 0.387 | 0.953 | 381 (225.05) | 0.488 | 0.101 | 0.387 | 0.868 | 0.949 |
| 2000 | 0.05 | 1294 (749.11) | 0.471 | 0.033 | 1930 (317.62) | 0.047 | 0.078 | 1223 (749.89) | 0.518 | 0.471 | 0.047 | 0.071 | 0.040 |
| 2000 | 0.50 | 1543 (690.96) | 0.305 | 0.408 | 1786 (525.37) | 0.143 | 0.518 | 1328 (746.31) | 0.448 | 0.305 | 0.143 | 0.434 | 0.492 |
| 2000 | 0.80 | 1702 (599.17) | 0.199 | 0.687 | 1610 (658.28) | 0.260 | 0.823 | 1312 (747.85) | 0.459 | 0.199 | 0.260 | 0.702 | 0.808 |
| 2000 | 0.95 | 1840 (463.90) | 0.107 | 0.869 | 1361 (742.11) | 0.426 | 0.962 | 1201 (748.74) | 0.533 | 0.107 | 0.426 | 0.871 | 0.960 |

**Table S6: Simulation Results Using Calibrated Upstrapping
Thresholds for 0.50 Stopping Point Monitoring**

|  | | ***Futility Only Monitoring*** | | | ***Efficacy Only Monitoring*** | | | ***Futility and Efficacy Monitoring*** | | | | | ***Fixed Sample*** |
| --- | --- | --- | --- | --- | --- | --- | --- | --- | --- | --- | --- | --- | --- |
| N | \(\beta\) | Expected N *mean(sd)* | Proportion Stopped | Proportion Rejected | Expected N *mean(sd)* | Proportion Stopped | Proportion Rejected | Expected N *mean(sd)* | Proportion Stopped | Proportion Stopped (Futility) | Proportion Stopped (Efficacy) | Proportion Rejected | Proportion Rejected |
| 40 | 0.05 | 26 (8.74) | 0.743 | 0.031 | 40 (4.10) | 0.044 | 0.070 | 25 (8.19) | 0.787 | 0.743 | 0.044 | 0.067 | 0.034 |
| 40 | 0.50 | 33 (9.62) | 0.363 | 0.353 | 35 (9.14) | 0.297 | 0.452 | 27 (9.48) | 0.660 | 0.363 | 0.297 | 0.423 | 0.382 |
| 40 | 0.80 | 37 (7.90) | 0.193 | 0.646 | 30 (10.00) | 0.506 | 0.733 | 27 (9.18) | 0.699 | 0.193 | 0.506 | 0.695 | 0.684 |
| 40 | 0.95 | 39 (5.04) | 0.068 | 0.886 | 26 (8.92) | 0.726 | 0.932 | 25 (8.09) | 0.794 | 0.068 | 0.726 | 0.903 | 0.915 |
| 160 | 0.05 | 102 (35.62) | 0.728 | 0.033 | 156 (17.44) | 0.050 | 0.075 | 98 (33.26) | 0.778 | 0.728 | 0.050 | 0.070 | 0.038 |
| 160 | 0.50 | 130 (38.91) | 0.383 | 0.359 | 138 (35.94) | 0.280 | 0.466 | 107 (37.83) | 0.663 | 0.383 | 0.280 | 0.416 | 0.409 |
| 160 | 0.80 | 146 (30.41) | 0.175 | 0.672 | 120 (40.02) | 0.504 | 0.763 | 106 (37.37) | 0.679 | 0.175 | 0.504 | 0.714 | 0.721 |
| 160 | 0.95 | 154 (21.21) | 0.076 | 0.876 | 103 (36.09) | 0.716 | 0.928 | 97 (32.49) | 0.792 | 0.076 | 0.716 | 0.888 | 0.916 |
| 600 | 0.05 | 378 (131.49) | 0.741 | 0.022 | 586 (64.79) | 0.049 | 0.069 | 363 (122.25) | 0.790 | 0.741 | 0.049 | 0.063 | 0.028 |
| 600 | 0.50 | 490 (144.75) | 0.368 | 0.418 | 501 (141.24) | 0.331 | 0.549 | 391 (137.68) | 0.699 | 0.368 | 0.331 | 0.478 | 0.489 |
| 600 | 0.80 | 541 (119.83) | 0.199 | 0.710 | 435 (149.29) | 0.551 | 0.797 | 375 (129.97) | 0.750 | 0.199 | 0.551 | 0.734 | 0.773 |
| 600 | 0.95 | 577 (80.02) | 0.077 | 0.889 | 373 (128.37) | 0.759 | 0.959 | 350 (111.14) | 0.836 | 0.077 | 0.759 | 0.899 | 0.949 |
| 2000 | 0.05 | 1246 (430.89) | 0.754 | 0.031 | 1950 (218.05) | 0.050 | 0.076 | 1196 (397.17) | 0.804 | 0.754 | 0.050 | 0.067 | 0.040 |
| 2000 | 0.50 | 1611 (487.77) | 0.389 | 0.425 | 1679 (467.09) | 0.321 | 0.545 | 1290 (453.99) | 0.710 | 0.389 | 0.321 | 0.478 | 0.492 |
| 2000 | 0.80 | 1801 (399.45) | 0.199 | 0.716 | 1453 (498.04) | 0.547 | 0.840 | 1254 (435.52) | 0.746 | 0.199 | 0.547 | 0.748 | 0.808 |
| 2000 | 0.95 | 1924 (265.13) | 0.076 | 0.904 | 1234 (423.58) | 0.766 | 0.966 | 1158 (364.92) | 0.842 | 0.076 | 0.766 | 0.910 | 0.960 |

**Table S7: Simulation Results Using Calibrated Upstrapping
Thresholds for 0.75 Stopping Point Monitoring**

|  | | ***Futility Only Monitoring*** | | | ***Efficacy Only Monitoring*** | | | ***Futility and Efficacy Monitoring*** | | | | | ***Fixed Sample*** |
| --- | --- | --- | --- | --- | --- | --- | --- | --- | --- | --- | --- | --- | --- |
| N | \(\beta\) | Expected N *mean(sd)* | Proportion Stopped | Proportion Rejected | Expected N *mean(sd)* | Proportion Stopped | Proportion Rejected | Expected N *mean(sd)* | Proportion Stopped | Proportion Stopped (Futility) | Proportion Stopped (Efficacy) | Proportion Rejected | Proportion Rejected |
| 40 | 0.05 | 32 (3.25) | 0.880 | 0.031 | 40 (2.18) | 0.050 | 0.062 | 31 (2.55) | 0.930 | 0.880 | 0.050 | 0.059 | 0.034 |
| 40 | 0.50 | 36 (4.96) | 0.436 | 0.367 | 37 (4.90) | 0.399 | 0.467 | 32 (3.71) | 0.835 | 0.436 | 0.399 | 0.452 | 0.382 |
| 40 | 0.80 | 39 (3.99) | 0.198 | 0.662 | 34 (4.75) | 0.656 | 0.745 | 32 (3.53) | 0.854 | 0.198 | 0.656 | 0.723 | 0.684 |
| 40 | 0.95 | 40 (2.24) | 0.053 | 0.902 | 32 (3.38) | 0.869 | 0.933 | 31 (2.68) | 0.922 | 0.053 | 0.869 | 0.920 | 0.915 |
| 160 | 0.05 | 125 (13.14) | 0.877 | 0.035 | 159 (8.64) | 0.049 | 0.063 | 123 (10.48) | 0.926 | 0.877 | 0.049 | 0.060 | 0.038 |
| 160 | 0.50 | 142 (19.97) | 0.470 | 0.378 | 146 (19.19) | 0.358 | 0.468 | 127 (15.10) | 0.828 | 0.470 | 0.358 | 0.437 | 0.409 |
| 160 | 0.80 | 153 (15.98) | 0.199 | 0.692 | 135 (19.26) | 0.636 | 0.759 | 127 (14.85) | 0.835 | 0.199 | 0.636 | 0.730 | 0.721 |
| 160 | 0.95 | 158 (9.65) | 0.062 | 0.898 | 126 (14.17) | 0.853 | 0.932 | 124 (11.16) | 0.915 | 0.062 | 0.853 | 0.914 | 0.916 |
| 600 | 0.05 | 467 (46.96) | 0.890 | 0.022 | 593 (32.71) | 0.050 | 0.065 | 459 (35.64) | 0.940 | 0.890 | 0.050 | 0.059 | 0.028 |
| 600 | 0.50 | 538 (74.05) | 0.419 | 0.449 | 532 (74.77) | 0.458 | 0.554 | 469 (49.29) | 0.877 | 0.419 | 0.458 | 0.514 | 0.489 |
| 600 | 0.80 | 572 (59.23) | 0.193 | 0.737 | 494 (68.03) | 0.711 | 0.798 | 465 (44.21) | 0.904 | 0.193 | 0.711 | 0.762 | 0.773 |
| 600 | 0.95 | 593 (33.32) | 0.052 | 0.920 | 466 (45.62) | 0.897 | 0.965 | 458 (33.02) | 0.949 | 0.052 | 0.897 | 0.936 | 0.949 |
| 2000 | 0.05 | 1541 (136.49) | 0.919 | 0.029 | 1976 (106.94) | 0.048 | 0.068 | 1517 (89.36) | 0.967 | 0.919 | 0.048 | 0.057 | 0.040 |
| 2000 | 0.50 | 1775 (248.92) | 0.451 | 0.437 | 1769 (249.40) | 0.462 | 0.554 | 1544 (140.99) | 0.913 | 0.451 | 0.462 | 0.499 | 0.492 |
| 2000 | 0.80 | 1901 (199.35) | 0.198 | 0.746 | 1627 (217.76) | 0.746 | 0.848 | 1528 (115.02) | 0.944 | 0.198 | 0.746 | 0.786 | 0.808 |
| 2000 | 0.95 | 1972 (115.02) | 0.056 | 0.928 | 1546 (143.88) | 0.909 | 0.967 | 1518 (91.94) | 0.965 | 0.056 | 0.909 | 0.935 | 0.960 |

**Table S8: Simulation Results Using Calibrated Upstrapping
Thresholds for Sequential Monitoring**

|  | | ***Futility Only Monitoring*** | | | ***Efficacy Only Monitoring*** | | | ***Futility and Efficacy Monitoring*** | | | | | ***Fixed Sample*** |
| --- | --- | --- | --- | --- | --- | --- | --- | --- | --- | --- | --- | --- | --- |
| N | \(\beta\) | Expected N *mean(sd)* | Proportion Stopped | Proportion Rejected | Expected N *mean(sd)* | Proportion Stopped | Proportion Rejected | Expected N *mean(sd)* | Proportion Stopped | Proportion Stopped (Futility) | Proportion Stopped (Efficacy) | Proportion Rejected | Proportion Rejected |
| 40 | 0.05 | 19 (9.32) | 0.916 | 0.023 | 39 (4.41) | 0.077 | 0.089 | 18 (7.96) | 0.968 | 0.905 | 0.063 | 0.069 | 0.034 |
| 40 | 0.50 | 27 (13.10) | 0.582 | 0.297 | 33 (8.87) | 0.444 | 0.504 | 20 (8.54) | 0.941 | 0.566 | 0.375 | 0.396 | 0.382 |
| 40 | 0.80 | 32 (12.26) | 0.355 | 0.562 | 28 (8.79) | 0.694 | 0.766 | 21 (7.61) | 0.944 | 0.340 | 0.604 | 0.629 | 0.684 |
| 40 | 0.95 | 37 (8.63) | 0.136 | 0.837 | 24 (6.68) | 0.895 | 0.942 | 22 (5.64) | 0.970 | 0.133 | 0.837 | 0.859 | 0.915 |
| 160 | 0.05 | 74 (38.38) | 0.910 | 0.029 | 151 (29.41) | 0.108 | 0.121 | 68 (32.42) | 0.965 | 0.871 | 0.094 | 0.100 | 0.038 |
| 160 | 0.50 | 107 (50.58) | 0.591 | 0.308 | 125 (46.18) | 0.425 | 0.514 | 75 (35.95) | 0.943 | 0.564 | 0.379 | 0.410 | 0.409 |
| 160 | 0.80 | 128 (48.49) | 0.339 | 0.586 | 101 (47.24) | 0.688 | 0.788 | 74 (36.32) | 0.937 | 0.326 | 0.611 | 0.646 | 0.721 |
| 160 | 0.95 | 142 (41.02) | 0.181 | 0.795 | 80 (40.65) | 0.877 | 0.937 | 67 (31.87) | 0.967 | 0.180 | 0.787 | 0.808 | 0.916 |
| 600 | 0.05 | 272 (135.05) | 0.938 | 0.015 | 564 (110.59) | 0.114 | 0.124 | 248 (114.44) | 0.979 | 0.889 | 0.090 | 0.094 | 0.028 |
| 600 | 0.50 | 409 (192.21) | 0.551 | 0.355 | 444 (172.43) | 0.520 | 0.596 | 273 (128.42) | 0.957 | 0.522 | 0.435 | 0.452 | 0.489 |
| 600 | 0.80 | 475 (185.34) | 0.340 | 0.626 | 363 (168.56) | 0.750 | 0.820 | 261 (120.04) | 0.971 | 0.325 | 0.646 | 0.663 | 0.773 |
| 600 | 0.95 | 541 (144.71) | 0.155 | 0.824 | 290 (140.39) | 0.912 | 0.967 | 248 (110.40) | 0.982 | 0.150 | 0.832 | 0.843 | 0.949 |
| 2000 | 0.05 | 891 (447.73) | 0.937 | 0.025 | 1888 (359.98) | 0.099 | 0.117 | 811 (370.70) | 0.983 | 0.894 | 0.089 | 0.095 | 0.040 |
| 2000 | 0.50 | 1319 (651.66) | 0.572 | 0.354 | 1496 (563.07) | 0.514 | 0.590 | 887 (410.53) | 0.972 | 0.538 | 0.434 | 0.447 | 0.492 |
| 2000 | 0.80 | 1588 (615.61) | 0.339 | 0.625 | 1197 (549.99) | 0.775 | 0.865 | 862 (388.08) | 0.982 | 0.326 | 0.656 | 0.669 | 0.808 |
| 2000 | 0.95 | 1797 (488.44) | 0.158 | 0.833 | 934 (462.45) | 0.924 | 0.972 | 794 (355.29) | 0.989 | 0.153 | 0.836 | 0.844 | 0.960 |

## Tabular Results for Group Sequential Calibrated Upstrapping (GU)

This section (***Table S9***:***Table
S12***) includes tabular results summaries for the group
sequential calibrated upstrapping (GU) approach to interim monitoring.
For each sample size (\(N\)) and power
(\(\beta\)) based scenario three
evaluation metrics are reported: expected total sample size
(*Expected \(N\)*), proportion
of trials that stopped early at any interim stopping point
(*Proportion Stopped*), and proportion of trials that either
stopped early for efficacy or rejected the null hypothesis with the full
sample data (*Proportion Rejected*). All tables also include
fixed sample rejection rate results for comparison. ***Table
S9*** reports results for trials designed with a single
interim stopping point at 25% of the total sample size.
***Table S10*** reports results for trials designed
with a single interim stopping point at 50% of the total sample size.
***Table S11*** reports results for trials designed
with a single interim stopping point at 75% of the total sample size.
***Table S12*** reports results for trials designed
with sequential interim stopping points at 25%, 50%, and 75% of the
total sample size.

**Table S9: Simulation Results Using Group Sequential O’Brien
Fleming P Values and Calibrated Upstrapping Proportion Thresholds for
0.25 Stopping Point Monitoring**

|  | | ***Futility Only Monitoring*** | | | ***Efficacy Only Monitoring*** | | | ***Futility and Efficacy Monitoring*** | | | | | ***Fixed Sample*** |
| --- | --- | --- | --- | --- | --- | --- | --- | --- | --- | --- | --- | --- | --- |
| N | \(\beta\) | Expected N *mean(sd)* | Proportion Stopped | Proportion Rejected | Expected N *mean(sd)* | Proportion Stopped | Proportion Rejected | Expected N *mean(sd)* | Proportion Stopped | Proportion Stopped (Futility) | Proportion Stopped (Efficacy) | Proportion Rejected | Proportion Rejected |
| 40 | 0.05 | 40 (0.00) | 0 | 0.044 | 40 (5.04) | 0.029 | 0.057 | 40 (5.04) | 0.029 | 0 | 0.029 | 0.063 | 0.034 |
| 40 | 0.50 | 40 (0.00) | 0 | 0.472 | 38 (8.92) | 0.098 | 0.412 | 38 (8.92) | 0.098 | 0 | 0.098 | 0.438 | 0.382 |
| 40 | 0.80 | 40 (0.00) | 0 | 0.758 | 35 (11.22) | 0.168 | 0.702 | 35 (11.22) | 0.168 | 0 | 0.168 | 0.728 | 0.684 |
| 40 | 0.95 | 40 (0.00) | 0 | 0.925 | 33 (13.26) | 0.266 | 0.914 | 33 (13.26) | 0.266 | 0 | 0.266 | 0.924 | 0.915 |
| 160 | 0.05 | 160 (0.00) | 0 | 0.050 | 155 (25.92) | 0.049 | 0.078 | 155 (25.92) | 0.049 | 0 | 0.049 | 0.084 | 0.038 |
| 160 | 0.50 | 160 (0.00) | 0 | 0.469 | 142 (42.87) | 0.150 | 0.447 | 142 (42.87) | 0.150 | 0 | 0.150 | 0.449 | 0.409 |
| 160 | 0.80 | 160 (0.00) | 0 | 0.763 | 129 (52.92) | 0.264 | 0.739 | 129 (52.92) | 0.264 | 0 | 0.264 | 0.742 | 0.721 |
| 160 | 0.95 | 160 (0.00) | 0 | 0.933 | 113 (58.74) | 0.397 | 0.921 | 113 (58.74) | 0.397 | 0 | 0.397 | 0.922 | 0.916 |
| 600 | 0.05 | 600 (0.00) | 0 | 0.038 | 578 (98.12) | 0.050 | 0.065 | 578 (98.12) | 0.050 | 0 | 0.050 | 0.074 | 0.028 |
| 600 | 0.50 | 600 (0.00) | 0 | 0.512 | 531 (162.51) | 0.154 | 0.499 | 531 (162.51) | 0.154 | 0 | 0.154 | 0.526 | 0.489 |
| 600 | 0.80 | 600 (0.00) | 0 | 0.788 | 484 (196.99) | 0.258 | 0.759 | 484 (196.99) | 0.258 | 0 | 0.258 | 0.787 | 0.773 |
| 600 | 0.95 | 600 (0.00) | 0 | 0.956 | 427 (219.18) | 0.386 | 0.947 | 427 (219.18) | 0.386 | 0 | 0.386 | 0.957 | 0.949 |
| 2000 | 0.05 | 2000 (0.00) | 0 | 0.044 | 1928 (320.81) | 0.048 | 0.076 | 1928 (320.81) | 0.048 | 0 | 0.048 | 0.080 | 0.040 |
| 2000 | 0.50 | 2000 (0.00) | 0 | 0.531 | 1786 (525.37) | 0.143 | 0.499 | 1786 (525.37) | 0.143 | 0 | 0.143 | 0.536 | 0.492 |
| 2000 | 0.80 | 2000 (0.00) | 0 | 0.832 | 1613 (656.63) | 0.258 | 0.806 | 1613 (656.63) | 0.258 | 0 | 0.258 | 0.829 | 0.808 |
| 2000 | 0.95 | 2000 (0.00) | 0 | 0.968 | 1361 (742.11) | 0.426 | 0.958 | 1361 (742.11) | 0.426 | 0 | 0.426 | 0.964 | 0.960 |

**Table S10: Simulation Results Using Group Sequential O’Brien
Fleming P Values and Calibrated Upstrapping Proportion Thresholds for
0.50 Stopping Point Monitoring**

|  | | ***Futility Only Monitoring*** | | | ***Efficacy Only Monitoring*** | | | ***Futility and Efficacy Monitoring*** | | | | | ***Fixed Sample*** |
| --- | --- | --- | --- | --- | --- | --- | --- | --- | --- | --- | --- | --- | --- |
| N | \(\beta\) | Expected N *mean(sd)* | Proportion Stopped | Proportion Rejected | Expected N *mean(sd)* | Proportion Stopped | Proportion Rejected | Expected N *mean(sd)* | Proportion Stopped | Proportion Stopped (Futility) | Proportion Stopped (Efficacy) | Proportion Rejected | Proportion Rejected |
| 40 | 0.05 | 26 (8.79) | 0.739 | 0.035 | 40 (4.28) | 0.048 | 0.070 | 25 (8.19) | 0.787 | 0.739 | 0.048 | 0.069 | 0.034 |
| 40 | 0.50 | 33 (9.62) | 0.362 | 0.421 | 34 (9.19) | 0.302 | 0.452 | 27 (9.45) | 0.664 | 0.362 | 0.302 | 0.441 | 0.382 |
| 40 | 0.80 | 37 (7.80) | 0.187 | 0.700 | 30 (10.00) | 0.508 | 0.732 | 27 (9.21) | 0.695 | 0.187 | 0.508 | 0.710 | 0.684 |
| 40 | 0.95 | 39 (4.83) | 0.062 | 0.899 | 26 (8.83) | 0.735 | 0.931 | 25 (8.05) | 0.797 | 0.062 | 0.735 | 0.912 | 0.915 |
| 160 | 0.05 | 104 (36.64) | 0.701 | 0.044 | 157 (15.87) | 0.041 | 0.066 | 101 (35.02) | 0.742 | 0.701 | 0.041 | 0.066 | 0.038 |
| 160 | 0.50 | 132 (38.18) | 0.350 | 0.414 | 142 (33.48) | 0.226 | 0.446 | 114 (39.56) | 0.576 | 0.350 | 0.226 | 0.409 | 0.409 |
| 160 | 0.80 | 148 (28.97) | 0.155 | 0.717 | 125 (39.77) | 0.444 | 0.750 | 113 (39.23) | 0.599 | 0.155 | 0.444 | 0.713 | 0.721 |
| 160 | 0.95 | 156 (19.01) | 0.060 | 0.905 | 108 (37.89) | 0.661 | 0.925 | 103 (35.90) | 0.721 | 0.060 | 0.661 | 0.900 | 0.916 |
| 600 | 0.05 | 383 (134.02) | 0.725 | 0.030 | 589 (58.11) | 0.039 | 0.052 | 371 (127.45) | 0.764 | 0.725 | 0.039 | 0.054 | 0.028 |
| 600 | 0.50 | 494 (143.72) | 0.356 | 0.437 | 509 (138.32) | 0.306 | 0.514 | 402 (141.98) | 0.662 | 0.356 | 0.306 | 0.473 | 0.489 |
| 600 | 0.80 | 543 (117.99) | 0.191 | 0.717 | 441 (149.80) | 0.530 | 0.773 | 384 (134.62) | 0.721 | 0.191 | 0.530 | 0.737 | 0.773 |
| 600 | 0.95 | 578 (78.57) | 0.074 | 0.897 | 379 (131.98) | 0.738 | 0.950 | 357 (117.27) | 0.812 | 0.074 | 0.738 | 0.902 | 0.949 |
| 2000 | 0.05 | 1291 (454.45) | 0.709 | 0.036 | 1954 (209.59) | 0.046 | 0.069 | 1235 (424.21) | 0.765 | 0.719 | 0.046 | 0.065 | 0.040 |
| 2000 | 0.50 | 1645 (478.75) | 0.355 | 0.464 | 1689 (463.13) | 0.311 | 0.524 | 1329 (470.09) | 0.671 | 0.360 | 0.311 | 0.490 | 0.492 |
| 2000 | 0.80 | 1836 (370.46) | 0.164 | 0.753 | 1469 (499.29) | 0.531 | 0.822 | 1303 (459.79) | 0.697 | 0.166 | 0.531 | 0.768 | 0.808 |
| 2000 | 0.95 | 1938 (241.28) | 0.062 | 0.922 | 1260 (438.85) | 0.740 | 0.962 | 1197 (397.93) | 0.803 | 0.063 | 0.740 | 0.921 | 0.960 |

**Table S11: Simulation Results Using Group Sequential O’Brien
Fleming P Values and Calibrated Upstrapping Proportion Thresholds for
0.75 Stopping Point Monitoring**

|  | | ***Futility Only Monitoring*** | | | ***Efficacy Only Monitoring*** | | | ***Futility and Efficacy Monitoring*** | | | | | ***Fixed Sample*** |
| --- | --- | --- | --- | --- | --- | --- | --- | --- | --- | --- | --- | --- | --- |
| N | \(\beta\) | Expected N *mean(sd)* | Proportion Stopped | Proportion Rejected | Expected N *mean(sd)* | Proportion Stopped | Proportion Rejected | Expected N *mean(sd)* | Proportion Stopped | Proportion Stopped (Futility) | Proportion Stopped (Efficacy) | Proportion Rejected | Proportion Rejected |
| 40 | 0.05 | 32 (3.44) | 0.863 | 0.037 | 40 (2.18) | 0.050 | 0.063 | 31 (2.60) | 0.927 | 0.877 | 0.050 | 0.061 | 0.034 |
| 40 | 0.50 | 36 (4.93) | 0.413 | 0.438 | 37 (4.90) | 0.397 | 0.465 | 32 (3.75) | 0.831 | 0.434 | 0.397 | 0.467 | 0.382 |
| 40 | 0.80 | 39 (3.70) | 0.164 | 0.728 | 34 (4.79) | 0.645 | 0.744 | 32 (3.69) | 0.838 | 0.193 | 0.645 | 0.737 | 0.684 |
| 40 | 0.95 | 40 (2.03) | 0.043 | 0.914 | 32 (3.58) | 0.849 | 0.929 | 31 (2.99) | 0.901 | 0.052 | 0.849 | 0.921 | 0.915 |
| 160 | 0.05 | 126 (13.63) | 0.866 | 0.042 | 159 (8.12) | 0.043 | 0.057 | 124 (11.16) | 0.915 | 0.872 | 0.043 | 0.054 | 0.038 |
| 160 | 0.50 | 142 (19.97) | 0.469 | 0.413 | 146 (19.16) | 0.356 | 0.467 | 127 (15.17) | 0.826 | 0.470 | 0.356 | 0.438 | 0.409 |
| 160 | 0.80 | 153 (15.98) | 0.199 | 0.717 | 135 (19.28) | 0.634 | 0.756 | 127 (14.89) | 0.834 | 0.200 | 0.634 | 0.727 | 0.721 |
| 160 | 0.95 | 158 (9.65) | 0.062 | 0.910 | 127 (14.33) | 0.849 | 0.932 | 124 (11.40) | 0.911 | 0.062 | 0.849 | 0.915 | 0.916 |
| 600 | 0.05 | 467 (46.77) | 0.891 | 0.027 | 593 (31.76) | 0.047 | 0.054 | 463 (40.95) | 0.919 | 0.872 | 0.047 | 0.059 | 0.028 |
| 600 | 0.50 | 536 (74.36) | 0.433 | 0.457 | 533 (74.65) | 0.449 | 0.533 | 470 (49.97) | 0.873 | 0.424 | 0.449 | 0.507 | 0.489 |
| 600 | 0.80 | 571 (59.80) | 0.198 | 0.743 | 494 (68.30) | 0.707 | 0.780 | 465 (44.00) | 0.905 | 0.198 | 0.707 | 0.762 | 0.773 |
| 600 | 0.95 | 592 (34.51) | 0.056 | 0.921 | 466 (45.81) | 0.896 | 0.958 | 458 (31.76) | 0.953 | 0.057 | 0.896 | 0.932 | 0.949 |
| 2000 | 0.05 | 1544 (141.72) | 0.912 | 0.034 | 1979 (101.48) | 0.043 | 0.061 | 1521 (100.34) | 0.958 | 0.915 | 0.043 | 0.054 | 0.040 |
| 2000 | 0.50 | 1784 (247.80) | 0.432 | 0.467 | 1782 (248.07) | 0.436 | 0.531 | 1557 (158.38) | 0.887 | 0.450 | 0.437 | 0.496 | 0.492 |
| 2000 | 0.80 | 1909 (193.02) | 0.182 | 0.768 | 1637 (223.12) | 0.726 | 0.831 | 1539 (134.15) | 0.922 | 0.194 | 0.728 | 0.780 | 0.808 |
| 2000 | 0.95 | 1975 (109.03) | 0.050 | 0.938 | 1551 (150.74) | 0.899 | 0.963 | 1524 (105.87) | 0.953 | 0.054 | 0.899 | 0.937 | 0.960 |

**Table S12: Simulation Results Using Group Sequential O’Brien
Fleming P Values and Calibrated Upstrapping Proportion Thresholds for
Sequential Monitoring**

|  | | ***Futility Only Monitoring*** | | | ***Efficacy Only Monitoring*** | | | ***Futility and Efficacy Monitoring*** | | | | | ***Fixed Sample*** |
| --- | --- | --- | --- | --- | --- | --- | --- | --- | --- | --- | --- | --- | --- |
| N | \(\beta\) | Expected N *mean(sd)* | Proportion Stopped | Proportion Rejected | Expected N *mean(sd)* | Proportion Stopped | Proportion Rejected | Expected N *mean(sd)* | Proportion Stopped | Proportion Stopped (Futility) | Proportion Stopped (Efficacy) | Proportion Rejected | Proportion Rejected |
| 40 | 0.05 | 24 (6.67) | 0.894 | 0.032 | 39 (6.31) | 0.093 | 0.104 | 23 (5.38) | 0.966 | 0.876 | 0.090 | 0.097 | 0.034 |
| 40 | 0.50 | 32 (9.26) | 0.480 | 0.405 | 32 (10.70) | 0.460 | 0.517 | 24 (7.72) | 0.907 | 0.465 | 0.442 | 0.483 | 0.382 |
| 40 | 0.80 | 36 (7.87) | 0.243 | 0.685 | 27 (10.86) | 0.698 | 0.774 | 22 (8.03) | 0.924 | 0.244 | 0.680 | 0.725 | 0.684 |
| 40 | 0.95 | 39 (4.96) | 0.080 | 0.891 | 22 (9.26) | 0.885 | 0.940 | 20 (7.70) | 0.956 | 0.080 | 0.876 | 0.910 | 0.915 |
| 160 | 0.05 | 97 (27.32) | 0.888 | 0.040 | 152 (28.88) | 0.097 | 0.110 | 90 (24.70) | 0.949 | 0.857 | 0.092 | 0.101 | 0.038 |
| 160 | 0.50 | 126 (36.12) | 0.518 | 0.386 | 127 (45.36) | 0.411 | 0.506 | 94 (34.10) | 0.898 | 0.497 | 0.401 | 0.455 | 0.409 |
| 160 | 0.80 | 145 (29.67) | 0.244 | 0.689 | 104 (47.79) | 0.673 | 0.777 | 89 (37.89) | 0.896 | 0.233 | 0.663 | 0.720 | 0.721 |
| 160 | 0.95 | 155 (19.80) | 0.086 | 0.892 | 82 (42.03) | 0.867 | 0.934 | 77 (35.92) | 0.944 | 0.086 | 0.858 | 0.899 | 0.916 |
| 600 | 0.05 | 354 (93.60) | 0.920 | 0.024 | 566 (108.96) | 0.105 | 0.108 | 330 (86.84) | 0.962 | 0.867 | 0.095 | 0.101 | 0.028 |
| 600 | 0.50 | 473 (137.56) | 0.493 | 0.416 | 450 (171.11) | 0.507 | 0.571 | 333 (117.34) | 0.933 | 0.455 | 0.478 | 0.505 | 0.489 |
| 600 | 0.80 | 533 (119.10) | 0.262 | 0.697 | 367 (169.30) | 0.745 | 0.804 | 306 (119.08) | 0.961 | 0.246 | 0.715 | 0.739 | 0.773 |
| 600 | 0.95 | 575 (80.69) | 0.096 | 0.883 | 293 (142.58) | 0.909 | 0.962 | 272 (115.46) | 0.977 | 0.093 | 0.884 | 0.898 | 0.949 |
| 2000 | 0.05 | 1185 (312.85) | 0.921 | 0.032 | 1891 (359.01) | 0.095 | 0.111 | 1096 (275.15) | 0.974 | 0.881 | 0.093 | 0.100 | 0.040 |
| 2000 | 0.50 | 1577 (458.38) | 0.492 | 0.423 | 1511 (566.30) | 0.491 | 0.567 | 1110 (380.01) | 0.940 | 0.473 | 0.467 | 0.497 | 0.492 |
| 2000 | 0.80 | 1802 (376.82) | 0.233 | 0.725 | 1216 (560.02) | 0.754 | 0.847 | 1031 (405.57) | 0.958 | 0.231 | 0.727 | 0.757 | 0.808 |
| 2000 | 0.95 | 1927 (249.47) | 0.084 | 0.907 | 952 (481.28) | 0.910 | 0.967 | 887 (394.83) | 0.976 | 0.086 | 0.890 | 0.911 | 0.960 |

## Tabular Results for Group Sequential Approaches (OBF, PO)

This section (***Table S13***:***Table
S14***) includes tabular results summaries for the
traditional group sequential design based approaches to interim
monitoring including both O’Brien-Fleming (OBF) and Pocock (PO). For
each sample size (\(N\)) and power
(\(\beta\)) based scenario three
evaluation metrics are reported: expected total sample size
(*Expected \(N\)*), proportion
of trials that stopped early at any interim stopping point
(*Proportion Stopped*), and proportion of trials that either
stopped early for efficacy or rejected the null hypothesis with the full
sample data (*Proportion Rejected*). Both tables also include
fixed sample rejection rate results for comparison. ***Table
S13*** reports results for trials designed with
O’Brien-Fleming p-value boundaries for sequential interim stopping
points at 25%, 50%, and 75% of the total sample size. ***Table
S14*** reports results for trials designed with Pocock
p-value boundaries for sequential interim stopping points at 25%, 50%,
and 75% of the total sample size.

**Table S13: Simulation Results Using Group Sequential O’Brien
Fleming P Value Thresholds for Sequential Monitoring**

|  | | ***Futility Only Monitoring*** | | | ***Efficacy Only Monitoring*** | | | ***Futility and Efficacy Monitoring*** | | | | | ***Fixed Sample*** |
| --- | --- | --- | --- | --- | --- | --- | --- | --- | --- | --- | --- | --- | --- |
| N | \(\beta\) | Expected N *mean(sd)* | Proportion Stopped | Proportion Rejected | Expected N *mean(sd)* | Proportion Stopped | Proportion Rejected | Expected N *mean(sd)* | Proportion Stopped | Proportion Stopped (Futility) | Proportion Stopped (Efficacy) | Proportion Rejected | Proportion Rejected |
| 40 | 0.05 | 25 (6.68) | 0.898 | 0.032 | 40 (0.94) | 0.009 | 0.036 | 24 (6.49) | 0.908 | 0.899 | 0.009 | 0.032 | 0.034 |
| 40 | 0.50 | 32 (9.06) | 0.493 | 0.402 | 39 (4.18) | 0.156 | 0.385 | 30 (8.42) | 0.650 | 0.494 | 0.156 | 0.373 | 0.382 |
| 40 | 0.80 | 36 (7.43) | 0.251 | 0.683 | 36 (5.98) | 0.372 | 0.688 | 32 (7.57) | 0.632 | 0.260 | 0.372 | 0.658 | 0.684 |
| 40 | 0.95 | 39 (4.32) | 0.074 | 0.897 | 33 (6.55) | 0.645 | 0.913 | 31 (6.66) | 0.732 | 0.087 | 0.645 | 0.888 | 0.915 |
| 160 | 0.05 | 103 (28.78) | 0.864 | 0.042 | 160 (6.64) | 0.019 | 0.043 | 102 (27.33) | 0.891 | 0.872 | 0.019 | 0.043 | 0.038 |
| 160 | 0.50 | 131 (33.52) | 0.484 | 0.407 | 151 (19.99) | 0.215 | 0.428 | 121 (30.50) | 0.703 | 0.488 | 0.215 | 0.391 | 0.409 |
| 160 | 0.80 | 147 (26.47) | 0.223 | 0.704 | 138 (26.68) | 0.463 | 0.732 | 125 (28.50) | 0.686 | 0.225 | 0.461 | 0.690 | 0.721 |
| 160 | 0.95 | 156 (16.61) | 0.070 | 0.904 | 123 (28.83) | 0.712 | 0.918 | 118 (27.79) | 0.784 | 0.072 | 0.712 | 0.895 | 0.916 |
| 600 | 0.05 | 398 (107.90) | 0.855 | 0.032 | 599 (17.69) | 0.008 | 0.022 | 397 (106.53) | 0.863 | 0.855 | 0.008 | 0.027 | 0.028 |
| 600 | 0.50 | 514 (116.48) | 0.396 | 0.463 | 559 (78.44) | 0.243 | 0.471 | 472 (111.69) | 0.642 | 0.399 | 0.243 | 0.457 | 0.489 |
| 600 | 0.80 | 558 (95.88) | 0.184 | 0.738 | 505 (106.49) | 0.500 | 0.753 | 462 (110.74) | 0.688 | 0.188 | 0.500 | 0.737 | 0.773 |
| 600 | 0.95 | 591 (47.17) | 0.046 | 0.928 | 444 (111.51) | 0.745 | 0.944 | 433 (106.94) | 0.797 | 0.053 | 0.744 | 0.925 | 0.949 |
| 2000 | 0.05 | 1348 (357.24) | 0.851 | 0.035 | 1992 (68.43) | 0.016 | 0.043 | 1333 (340.31) | 0.880 | 0.864 | 0.016 | 0.038 | 0.040 |
| 2000 | 0.50 | 1724 (393.64) | 0.367 | 0.489 | 1855 (271.20) | 0.246 | 0.483 | 1571 (381.58) | 0.628 | 0.381 | 0.247 | 0.479 | 0.492 |
| 2000 | 0.80 | 1888 (284.67) | 0.149 | 0.782 | 1655 (364.71) | 0.533 | 0.804 | 1541 (366.68) | 0.687 | 0.154 | 0.533 | 0.776 | 0.808 |
| 2000 | 0.95 | 1975 (142.81) | 0.033 | 0.951 | 1439 (369.34) | 0.787 | 0.958 | 1417 (352.71) | 0.821 | 0.034 | 0.787 | 0.946 | 0.960 |

**Table S14: Simulation Results Using Group Sequential Pocock P
Value Thresholds for Sequential Monitoring**

|  | | ***Futility Only Monitoring*** | | | ***Efficacy Only Monitoring*** | | | ***Futility and Efficacy Monitoring*** | | | | | ***Fixed Sample*** |
| --- | --- | --- | --- | --- | --- | --- | --- | --- | --- | --- | --- | --- | --- |
| N | \(\beta\) | Expected N *mean(sd)* | Proportion Stopped | Proportion Rejected | Expected N *mean(sd)* | Proportion Stopped | Proportion Rejected | Expected N *mean(sd)* | Proportion Stopped | Proportion Stopped (Futility) | Proportion Stopped (Efficacy) | Proportion Rejected | Proportion Rejected |
| 40 | 0.05 | 17 (9.44) | 0.925 | 0.024 | 40 (1.92) | 0.012 | 0.020 | 16 (8.45) | 0.966 | 0.955 | 0.011 | 0.021 | 0.034 |
| 40 | 0.50 | 25 (13.61) | 0.618 | 0.312 | 38 (6.58) | 0.173 | 0.296 | 22 (11.44) | 0.835 | 0.678 | 0.157 | 0.261 | 0.382 |
| 40 | 0.80 | 30 (13.46) | 0.396 | 0.558 | 34 (8.68) | 0.394 | 0.572 | 24 (11.40) | 0.792 | 0.440 | 0.352 | 0.493 | 0.684 |
| 40 | 0.95 | 35 (11.41) | 0.209 | 0.777 | 30 (9.22) | 0.664 | 0.828 | 24 (9.94) | 0.845 | 0.230 | 0.615 | 0.735 | 0.915 |
| 160 | 0.05 | 78 (39.34) | 0.896 | 0.038 | 158 (14.40) | 0.030 | 0.036 | 69 (35.07) | 0.956 | 0.928 | 0.028 | 0.035 | 0.038 |
| 160 | 0.50 | 112 (49.20) | 0.559 | 0.355 | 144 (33.28) | 0.240 | 0.355 | 86 (43.74) | 0.849 | 0.630 | 0.219 | 0.306 | 0.409 |
| 160 | 0.80 | 132 (45.98) | 0.313 | 0.628 | 126 (41.00) | 0.496 | 0.652 | 90 (43.03) | 0.844 | 0.385 | 0.459 | 0.557 | 0.721 |
| 160 | 0.95 | 145 (37.53) | 0.153 | 0.828 | 105 (42.56) | 0.730 | 0.866 | 86 (40.11) | 0.881 | 0.203 | 0.678 | 0.770 | 0.916 |
| 600 | 0.05 | 316 (148.35) | 0.888 | 0.028 | 590 (64.32) | 0.031 | 0.035 | 282 (125.55) | 0.964 | 0.936 | 0.028 | 0.033 | 0.028 |
| 600 | 0.50 | 450 (178.65) | 0.474 | 0.427 | 526 (134.40) | 0.284 | 0.398 | 350 (161.64) | 0.820 | 0.553 | 0.267 | 0.360 | 0.489 |
| 600 | 0.80 | 506 (167.34) | 0.268 | 0.676 | 457 (160.61) | 0.531 | 0.680 | 344 (159.44) | 0.842 | 0.341 | 0.501 | 0.619 | 0.773 |
| 600 | 0.95 | 561 (118.40) | 0.107 | 0.874 | 369 (162.05) | 0.771 | 0.902 | 320 (148.03) | 0.891 | 0.153 | 0.738 | 0.822 | 0.949 |
| 2000 | 0.05 | 1085 (497.35) | 0.877 | 0.039 | 1966 (203.43) | 0.032 | 0.039 | 996 (432.34) | 0.947 | 0.915 | 0.032 | 0.042 | 0.040 |
| 2000 | 0.50 | 1501 (602.38) | 0.465 | 0.454 | 1717 (486.98) | 0.295 | 0.408 | 1176 (547.10) | 0.793 | 0.506 | 0.287 | 0.381 | 0.492 |
| 2000 | 0.80 | 1742 (517.88) | 0.223 | 0.727 | 1458 (554.75) | 0.568 | 0.726 | 1168 (520.14) | 0.833 | 0.261 | 0.572 | 0.679 | 0.808 |
| 2000 | 0.95 | 1884 (383.18) | 0.090 | 0.902 | 1186 (527.84) | 0.804 | 0.921 | 1064 (484.20) | 0.894 | 0.112 | 0.782 | 0.867 | 0.960 |

## AU Single Stopping Point Data Visualizations

This section includes graphical results summaries comparing between
the various versions of arbitrary upstrapping (AU) presented in
***Table S5***:***Table S8***.
For each sample size (\(N\), shown on
the y axis) and power (\(\beta\), shown
on the x axis) based scenario three evaluation metrics are reported:
expected total sample size (*Expected Sample Size*), proportion
of trials that either stopped early for efficacy or rejected the null
hypothesis with the full sample data (*Rejection Rate*), and
proportion of trials that stopped early at any interim stopping point
(*Interim Stopping Rate*). The *AU* method refers to
trials designed with sequential stopping points at 25%, 50%, and 75% of
the total sample size. The *AU 0.25* method refers to trials
designed with a single interim stopping point at 25% of the total sample
size. The *AU 0.50* method refers to trials designed with a
single interim stopping point at 50% of the total sample size. The
*AU 0.75* method refers to trials designed with a single interim
stopping point at 75% of the total sample size.

**Figure S1:** AU single stopping point results

## CU Single Stopping Point Data Visualizations

This section includes graphical results summaries comparing between
the various versions of calibrated upstrapping (CU) presented in
***Table S1***:***Table S4***.
For each sample size (\(N\), shown on
the y axis) and power (\(\beta\), shown
on the x axis) based scenario three evaluation metrics are reported:
expected total sample size (*Expected Sample Size*), proportion
of trials that either stopped early for efficacy or rejected the null
hypothesis with the full sample data (*Rejection Rate*), and
proportion of trials that stopped early at any interim stopping point
(*Interim Stopping Rate*). The *CU* method refers to
trials designed with sequential stopping points at 25%, 50%, and 75% of
the total sample size. The *CU 0.25* method refers to trials
designed with a single interim stopping point at 25% of the total sample
size. The *CU 0.50* method refers to trials designed with a
single interim stopping point at 50% of the total sample size. The
*CU 0.75* method refers to trials designed with a single interim
stopping point at 75% of the total sample size.

**Figure S2:** CU single stopping point results

## GU Single Stopping Point Data Visualizations

This section includes graphical results summaries comparing between
the various versions of group sequential calibrated upstrapping (GU)
presented in ***Table S9***:***Table
S12***. For each sample size (\(N\), shown on the y axis) and power (\(\beta\), shown on the x axis) based
scenario three evaluation metrics are reported: expected total sample
size (*Expected Sample Size*), proportion of trials that either
stopped early for efficacy or rejected the null hypothesis with the full
sample data (*Rejection Rate*), and proportion of trials that
stopped early at any interim stopping point (*Interim Stopping
Rate*). The *GU* method refers to trials designed with
sequential stopping points at 25%, 50%, and 75% of the total sample
size. The *GU 0.25* method refers to trials designed with a
single interim stopping point at 25% of the total sample size. The
*GU 0.50* method refers to trials designed with a single interim
stopping point at 50% of the total sample size. The *GU 0.75*
method refers to trials designed with a single interim stopping point at
75% of the total sample size.

**Figure S3:** GU single stopping point results

## Combined Data Visualizations

This section includes graphical results summaries comparing
simultaneously between all the various versions of upstrapping (CU, AU,
or GU) presented in ***Table S1***:***Table
S12***. For each sample size (\(N\), shown on the y axis) and power (\(\beta\), shown on the x axis) based
scenario three evaluation metrics are reported: expected total sample
size (*Expected Sample Size*), proportion of trials that either
stopped early for efficacy or rejected the null hypothesis with the full
sample data (*Rejection Rate*), and proportion of trials that
stopped early at any interim stopping point (*Interim Stopping
Rate*). The *CU, AU, GU* methods refer to trials designed
with sequential stopping points at 25%, 50%, and 75% of the total sample
size. The *CU 0.25, AU 0.25, GU 0.25* methods refer to trials
designed with a single interim stopping point at 25% of the total sample
size. The *CU 0.50, AU 0.50, GU 0.50* methods refer to trials
designed with a single interim stopping point at 50% of the total sample
size. The *CU 0.75, AU 0.75, GU 0.75* methods refer to trials
designed with a single interim stopping point at 75% of the total sample
size.

**Figure S4:** Combined results for all methods

## Sensitivity Analysis: Upstrapping Results Excluding the 25% Monitoring Point

Due to concerns that method performance might be inhibited by lack of
sufficient data at particularly early monitoring points, simulations
dropping the earliest interim analysis (at 25% of the full sample) were
implemented. Results from this sensitivity analysis are consistent with
the main results findings overall, with small to moderate gains in
operating characteristics across methods. Without the possibility of
stopping for futility at 25% of the full sample ESS increased for all
methods proportionally.

While CP 1% still performs best out of all four CP based methods, the
decrease in power under the alternative is lessened compared to the main
results findings, with power reductions of up to 1.6% and 7.2% for CP 1%
and CP 5% respectively. Type I error rate was consistent with previous
results. For ESS the difference between the four CP methods was
narrower, ranging from 52-65% of FS under the null and 83-100% under the
alternative.

AU and CU both showed significant gains in power after excluding the
first interim look, with power reductions between 1.2-3.3% compared to
FS for AU and 5.2-11.6% for CU. Type I error rate showed slight
improvement, remaining within 0.5% of FS for AU and within 1.3% for CU.
Under the null scenario ESS was around 69% of FS for AU and around 59%
for CU. For the alternative ESS was approximately 95% of FS for AU and
89% for CU. Results remain unchanged for GU since it’s specific
calibration approach already excludes the first interim monitoring
point.

Excluding the first interim look does have a positive effect on
performance of the upstrapping based designs. Both AU and CU achieved
improvements in power and type I error rate and higher ESS under the
alternative scenario. While ESS under the null scenario showed more
modest results compared to the main findings, it was still significantly
reduced compared to FS. After excluding the first interim look the
upstrapping designs were more comparable to both OBF, CP 5%, and CP
1%.

The full results of this sensitivity analysis are shown below in
***Table S15*** and Figure S5 below.

***Table S15*** describes sensitivity analysis
results from alternative and null scenario simulations for the
performance of the interim analysis calibration method, excluding the
first interim monitoring point, relative to the fixed sample design. CU
is the calibrated upstrap, AU is the arbitrary upstrap, GU is the
group-sequential upstrap, OBF is the O’Brien-Fleming alpha-spending
function, PO is the Pocok alpha-spending function, CP is the conditional
power method. ESS refers here to expected sample size, while TIE refers
to the type I error rate. Any positive increases in TIE and power for
GU, OBF, and PO relative to the fixed sample occur because group
sequential designs involve an adjusted p-value boundary for the final
analysis.

**Table S15: Sensitivity Analysis Results with Two Stopping Points
(50% and 75%)**

|  | | ***Null*** | | ***Alternative*** | |
| --- | --- | --- | --- | --- | --- |
| Method | N | Expected N Ratio | Type I Error Difference | Expected N Ratio | Power Difference |
| 0.50-0.75 AU | 40 | 0.68 | 0.000 | 0.95 | -0.016 |
| 0.50-0.75 AU | 160 | 0.68 | -0.002 | 0.94 | -0.020 |
| 0.50-0.75 AU | 600 | 0.69 | -0.004 | 0.94 | -0.026 |
| 0.50-0.75 AU | 2000 | 0.70 | -0.005 | 0.95 | -0.033 |
| 0.50-0.75 CU | 40 | 0.60 | -0.006 | 0.90 | -0.052 |
| 0.50-0.75 CU | 160 | 0.59 | -0.005 | 0.89 | -0.065 |
| 0.50-0.75 CU | 600 | 0.58 | -0.009 | 0.88 | -0.080 |
| 0.50-0.75 CU | 2000 | 0.58 | -0.013 | 0.88 | -0.116 |
| 0.50-0.75 GU | 40 | 0.60 | -0.002 | 0.90 | 0.001 |
| 0.50-0.75 GU | 160 | 0.61 | 0.002 | 0.91 | -0.032 |
| 0.50-0.75 GU | 600 | 0.59 | -0.004 | 0.89 | -0.076 |
| 0.50-0.75 GU | 2000 | 0.59 | -0.008 | 0.90 | -0.083 |
| 0.50-0.75 CP 1% | 40 | 0.65 | -0.013 | 1.00 | -0.002 |
| 0.50-0.75 CP 1% | 160 | 0.63 | -0.020 | 0.96 | -0.008 |
| 0.50-0.75 CP 1% | 600 | 0.63 | -0.012 | 0.96 | -0.008 |
| 0.50-0.75 CP 1% | 2000 | 0.62 | -0.019 | 0.97 | -0.016 |
| 0.50-0.75 CP 5% | 40 | 0.58 | -0.017 | 0.98 | -0.010 |
| 0.50-0.75 CP 5% | 160 | 0.58 | -0.021 | 0.92 | -0.032 |
| 0.50-0.75 CP 5% | 600 | 0.56 | -0.015 | 0.91 | -0.059 |
| 0.50-0.75 CP 5% | 2000 | 0.56 | -0.024 | 0.92 | -0.072 |
| 0.50-0.75 CP 10% | 40 | 0.55 | -0.020 | 0.92 | -0.082 |
| 0.50-0.75 CP 10% | 160 | 0.54 | -0.026 | 0.90 | -0.061 |
| 0.50-0.75 CP 10% | 600 | 0.54 | -0.020 | 0.88 | -0.100 |
| 0.50-0.75 CP 10% | 2000 | 0.54 | -0.029 | 0.88 | -0.123 |
| 0.50-0.75 CP 20% | 40 | 0.55 | -0.024 | 0.88 | -0.119 |
| 0.50-0.75 CP 20% | 160 | 0.52 | -0.031 | 0.84 | -0.140 |
| 0.50-0.75 CP 20% | 600 | 0.52 | -0.024 | 0.83 | -0.181 |
| 0.50-0.75 CP 20% | 2000 | 0.52 | -0.033 | 0.83 | -0.220 |

***Figure S5*** describes the results for a
sensitivity analysis excluding the first interim monitoring point
(i.e. interim analyses are performed at 50% and 75% of the total planned
sample). The panels from left to right show mean expected sample size,
rejection rate, and interim stopping rate.

**Figure S5:** Sensitivity Analysis Results
